# Supplementary material for: Substrate recognition and cryo-EM structure of the ribosome-bound TAC toxin of Mycobacterium tuberculosis
Source: Nat Commun. 2022 May 12;13:2641. doi: 10.1038/s41467-022-30373-w (PMC9098466; doi:10.1038/s41467-022-30373-w)

SUPPLEMENTARY INFORMATION FILE

Substrate recognition and cryo-EM structure of the ribosome-bound TAC toxin of *Mycobacterium tuberculosis*

Moise Mansour, Emmanuel Giudice, Xibing Xu, Hatice Akarsu, Patricia Bordes, Valérie Guillet, Donna-Joe Bigot, Nawel Slama, Gaetano D'urso, Sophie Chat, Peter Redder, Laurent Falquet, Lionel Mourey, Reynald Gillet, Pierre Genevaux

This PDF file includes:

Supplementary Methods: Plasmid constructs

Supplementary Fig. 1 to 13

Supplementary Table 1 to 3

Uncropped scans

Other Supplementary Materials for this manuscript include the following:

Supplementary Data 1 contains nEMOTE data as a separate excel file

Supplementary Methods

Plasmid constructs

Plasmids pMPMK6[1], pET-20b (Novagen), pET-20b-HigB^{TAC} and pET-20b-HigB^{TAC}[K95A][2] pBAD33[3], pK6-HigB^{TAC} [4], pK6-GFP[5], pSE380ΔNcoI[6], pLAM12[7], pGMC[8] and pGMC-TAC[2] have been described.

All the primers used in this study are listed in **Supplementary Table 3**. To construct plasmids pGMC-HigB^{TAC}, pGMC-HigB2 and pGMC-HigB3, *higB^{TAC}* (Rv1955; 378pb), *higB2* (Rv2022c; 444pb) and *higB3* (Rv3182; 345pb) were PCR amplified from *M. tuberculosis* H37Rv genomic DNA using primers HigB1_pGMC_Fw and HigB1_pGMC_Rv, HigB2_pGMC_Fw and HigB2_pGMC_Rv or HigB3_pGMC_Fw and HigB3_pGMC_Rv, respectively. Compared to the first genome annotation, a shorter and more accurate version of *higB2* based on the analysis of available RNAseq and ribosome profiling data for *M. tuberculosis* (https://www.wadsworth.org/research/scientific-resources/interactive-genomics) was used in our experiments. In this case, HigB2 protein sequence is 148 amino acid long, starting at the sequence MSYPEEYH. PCR fragments were cloned into the pGMC plasmid linearized using pGMC_Infusion_Fw and pGMC_Infusion_Rv primers with the In-Fusion PCR cloning system. R68A, R77A and K95A substitutions in pGMC-HigB^{TAC} were obtained by QuickChange site directed mutagenesis using pGMC-HigB^{TAC} as DNA template and primers HigB1_R68A_pGMC_Fw and HigB1_R68A_pGMC_Rv, HigB1_R77A_pGMC_Fw and HigB1_R77A_pGMC_Rv or HigB1_K95A_pGMC_Fw and HigB1_K95A_pGMC_Rv respectively. K58A, E66A, N73A, Q98A, K99A, R61A, E106A, D110A, K113A and R117A substitutions in pGMC-HigB^{TAC} were constructed by InFusion using pK6-HigB^{TAC} alanine mutations as template and with primers HigB1_pGMC_Fw and HigB1_pGMC_Rv.

To construct pLAM12-HigB2 and pLAM12-HigB3, PCR fragments of *higB2*, *higB3* were subcloned from, pGMC-HigB2 or pGMC-HigB3 using primers HigB2_pLAM_Fw and

HigB2_pLAM_Rv, and HigB3_pLAM_Fw and HigB3_pLAM_Rv, respectively, and cloned by In-Fusion PCR cloning system into pLAM12 vector linearized with primers pLAM_Infusion_Fw and pLAM_Infusion_Rv. To construct pLAM12-HigB^{TAC} and pLAM12- HigB^{TAC}[K95A], *higB*^{TAC} PCR fragments were subcloned from *M. tuberculosis* H37Rv genomic DNA using primers HigB1_pLAM_Fw and HigB1_pLAM_Rv and cloned by In-Fusion PCR cloning system into pLAM12 vector linearized using primers pLAM_Infusion_Fw and pLAM_Infusion_Rv. K95A substitution was obtained by QuickChange site directed mutagenesis using pLAM-HigB^{TAC} as DNA template and primers HigB1_K95A_pLAM_Fw and HigB1_K95A_pLAM_Rv.

For pET20b-HigB2 and -HigB3, *higB2* and *higB3* PCR fragment were subcloned from pGMC-*Mtb*-HigB2 and pGMC-*Mtb*-HigB3 using primers higB2_infusion_20b_for and higB2_infusion_20b_rev, and HigB3_ infusion_20b_for and HigB3_ infusion_20b_rev, respectively, and cloned by In-Fusion into pET20b vector linearized using primer pET20b_Infusion_Fw and pET20b_Infusion_Rv. To construct pET20b-HigB^{TAC} with K61A, and K113A substitutions in pET20b-HigB^{TAC}, the corresponding alleles were PCR amplified from pK6-HigB^{TAC} plasmids using primers HigB1-pET20b-NdeI-Fw and HigB1-pET20b-XhoI-Rv, and cloned as an NdeI/XhoI fragment into pET20b vector. For pET15b-HigB^{TAC}-K95Aopt, PCR fragment using primers HigB1opt_NdeI_Fw and HigB1opt_BamHI_Rv, and pET20b-HigB^{TAC} K95A as template was cloned as an NdeI/BamHI fragment into pET15b vector digested with the same enzymes.

To construct plasmid pSE380ΔNcoI-CspA, the *cspA* gene (204 bp) was PCR amplified from *M. tuberculosis* H37Rv genomic DNA using primers CspA_IF_Fw and CspA_IF_Rv, and cloned by In-Fusion PCR cloning system into pSE380ΔNcoI linearized using primers pSE_Infusion_Fw and pSE_Infusion_Rv. Plasmids pSE-CspA with CCA codons at position 12, 17, 22, 27, 39, 44, 49 and 54, and with the CCA at position Pro2

replaced by CCG codon were obtained by QuickChange site directed mutagenesis using pSE-*Mtb*-CspA as template with the primers CspA_codon 12_Fw and CspA_codon 12_Rv, CspA_codon 17_Fw and CspA_codon 17_Rv, CspA_codon 22_Fw and CspA_codon 22_Rv, CspA_codon 27_Fw and CspA_codon 27_Rv, CspA_codon 39_Fw and CspA_codon 39_Rv, CspA_codon 44_Fw and CspA_codon 44_Rv, CspA_codon 49_Fw and CspA_codon 49_Rv, CspA_codon 54_Fw and CspA_codon 54_Rv, respectively. For plasmid pSE380ΔNcoI-GroES with a c-terminal Strep Tag, the *groES* gene (303bp) was amplified from *M. tuberculosis* H37Rv genomic DNA using primers GroES_IF_Fw and GroES-strep_IF_Rv, and cloned by In-Fusion PCR cloning system into linearized pSE380ΔNcoI.

To construct pBAD33-HigB^{TAC}, EcoRV/SphI digested *higB* from pK6-HigB^{TAC} was ligated into pBAD33 digested with the same enzymes. For plasmids pBAD33-HigB2 and -HigB3, *higB2* and *higB3* were PCR amplified from *M. tuberculo*sis H37Rv genome using primers HigB2_pBAD33_Fw and HigB2_pBAD33_Rv, or HigB3_pBAD33_Fw and HigB3_pBAD33_Rv, respectively, and cloned by In-Fusion into pBAD33 vector linearized with pBAD33_Infusion_Fw/ pBAD33_Infusion_Rv. HigB^{TAC} alanine substitutions in pK6-HigB^{TAC} plasmid were obtained by quickchange mutagenesis using appropriate primers (**Supplementary Table 3**). All the plasmids were sequence-verified.

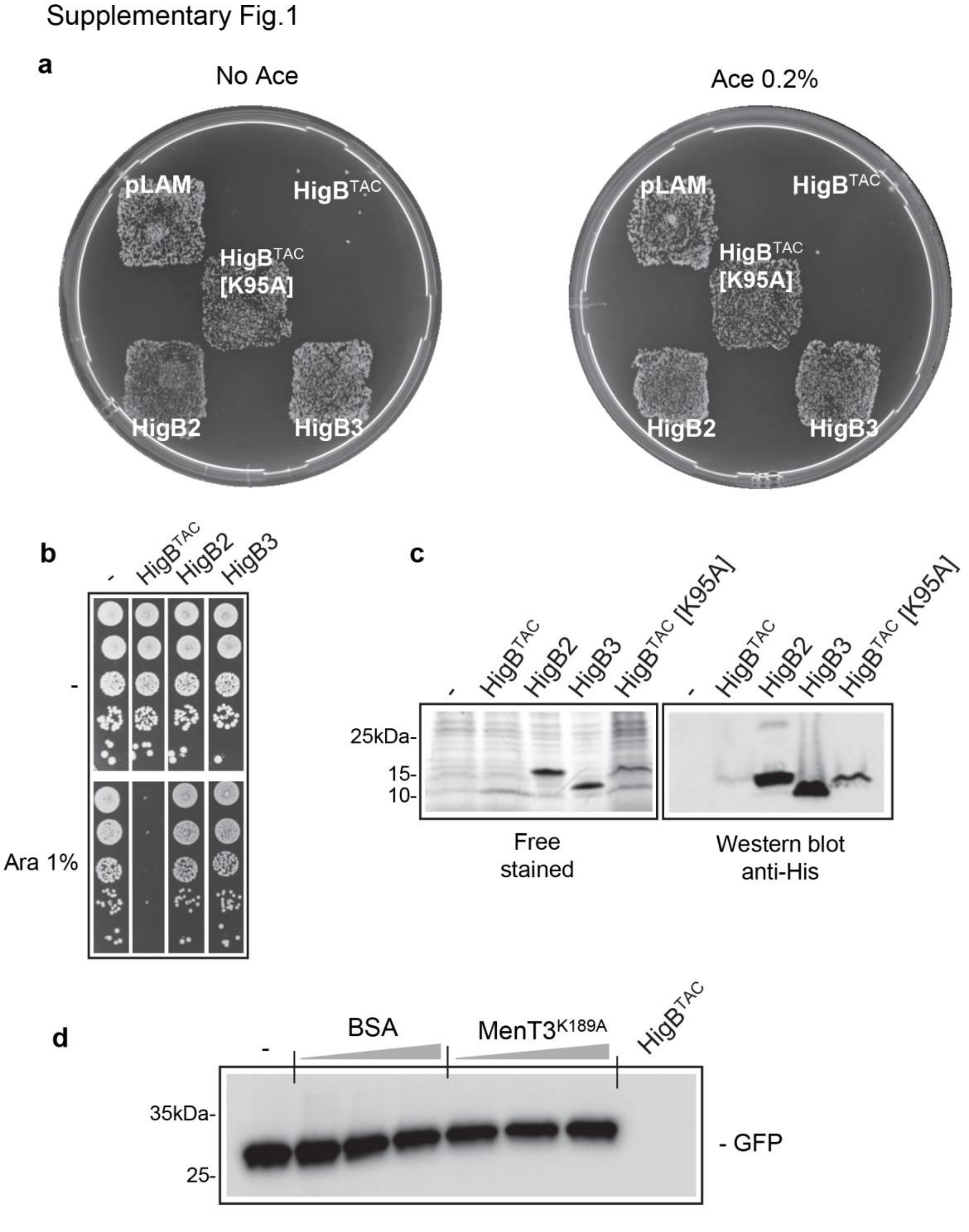

Supplementary Fig. 1: HigB^{TAC}, but not HigB2 and HigB3, impacts *M. smegmatis* and *E. coli* growth. **a** Cultures of *M. smegmatis* transformed with pLAM-vector, HigB^{TAC}, HigB^{TAC} [K95A], HigB2 or HigB3 were plated on LB kanamycin agar plates with or without acetamide (Ace) inducer at the indicated concentration. Plates were incubated 3 days at 37

°C. Representative results of two independent experiments are shown. **b** Cultures of *E. coli* transformed with pBAD33-vector (-), $HigB^{TAC}$, HigB2 or HigB3 were serial diluted and spotted on LB agar plates in the absence or presence of 1% arabinose inducer (Ara). Plates were incubated overnight at 37 °C. Representative results of three independent experiments are shown. **c** Steady state levels of His-tagged $HigB^{TAC}$, $HigB^{TAC}$ [K95A], HigB2 and HigB3 in pET20b expressed in BL21AI in the presence of 0.2% arabinose inducer overnight at 25 °C. Free stained SDS-PAGE of whole cell extracts (left) and the corresponding western blot using anti-His antibodies (right) are shown. Note that the signal obtained for HigB2 by western blot with anti-His antibodies was very strong when compared to the other two toxins. Therefore, the whole cell extract of the HigB2 sample was diluted 50 fold for the western blot. Representative results of two independent experiments are shown. **d** *In vitro* transcription/translation reactions assessing the synthesis of GFP protein in the absence (-) or presence of increasing concentrations of BSA or MenT3[K189A] (0.3, 3 and 6 µM). Samples were separated by SDS-PAGE and revealed by western blot using an anti-GFP antibody. Representative results of three independent experiments are shown.

Supplementary Fig.2

a

toxins	genes	position (codon)	motif	genomic position	cleavages/ replicates	Mtb: transcript/ cleavage site	essentiality Msmeg/Mtb	functional annotation
**HigB**^{TAC}	*MSMEG_1076*	23/91	CC/A	+1141497	267/6	-/-	-/NA	Hypothetical protein
	groES	8/100	CC/A	+1671201	183/6	+/+	-/+	Protein folding
	cspA	2/67	CC/A	-6230161	399/5	+/+	-/-	RNA chaperone
	rpsA	2/479	CC/A	-3904814	156/5	+/-	+/+	Translation
	ssb	36/165	AC/A	-6942744	57/5	+/-	+/+	Replication
	usfY	14/99	CC/A	+1858535	187/4	-/-	-/ NA	LPS metabolic process
	nat1	NA	UU/A	-5862601	65/4	NA/NA	NA	NA
	MSMEG_1770	6/80	UC/A	-1859100	65/4	-/-	NA	Hypothetical protein
	nat2	NA	UU/A	+354	28/3	NA/NA	NA	NA
	rpsL[b]	74/124	CU/U	+1498523	27/3	+/-	+/+	Translation
	parA	300/323	UC/A	-6981819	31/3	+/-	-/+	Replication
	MSMEG_5607	53/82	CC/A	+5693295	30/3	-/-	-/ NA	Hypothetical protein
	nat3	NA	UG/A	+5184433	33/3	NA/NA	NA	NA
	MSMEG_1773[b]	42/340	CC/A	-1861165	85/2	-/-	-/ NA	Hypothetical protein
	MSMEG_1773[a]	19/340	AU/A	-1861234	61/2	-/-	-/ NA	Hypothetical protein
	rpsL[a]	2/124	CC/A	+1498307	206/3	+/+	+/+	Translation
	nat4	NA	AC/A	-5862595	30/2	NA/NA	NA	NA
	MSMEG_0595	25/245	GC/A	+673478	24/2	-/-	-/ NA	Hypothetical protein
	MSMEG_2727	206/273	CC/A	-2795804	20/2	-/-	-/ NA	Hypothetical protein
	infC	201/206	CC/A	-3861748	19/2	+/-	+/+	Translation
	mce2A	392/404	GU/G	+5884998	19/2	-/-	-/ NA	Virulence, adaptation
	rplJ	140/175	AU/G	+1459087	16/2	+/+	-/+	Translation
	glpK	15/505	AC/A	+6803807	15/2	-/-	-/ NA	Metabolism
	MSMEG_0702	261/310	UC/A	-790771	14/2	-/-	-/ NA	Hypothetical protein
**HigB**^{TAC}**K95A**	nat5	NA	UU/G	+307135	22/2	NA/NA	NA	NA
**HigB2** (24h)	*cdd*	34/128	GAC/GAC	+1766889	60/2	+/-	-/-	Metabolism
	yajC	28/107	GCC/ATG	+3022816	43/2	+/+	-/-	Secretion
	nat6	NA	UC/C	+6371128	42/2	NA/NA	NA	NA
**HigB3** (24h)	*yajC*	28/107	GCC/ATG	+3022816	22/2	+/+	-/-	Secretion

b

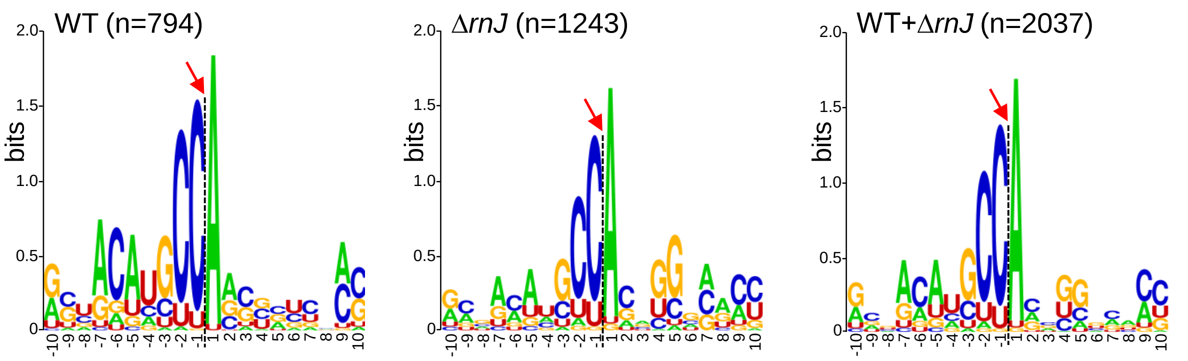

c

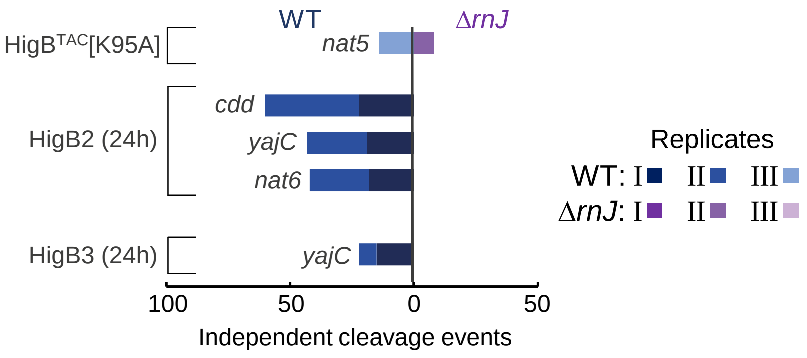

Supplementary Fig. 2: Cleavage sites and overview of the targets identified. a General information about the cleavage sites identified in this work. "NA" stands for not available or non-applicable. The gene names or the locus tags are indicated when available. Names nat1 to nat6 stand for previously non-annotated transcripts that have been found by RNAseq and ribosome profiling of the *M. smegmatis* genome available at https://www.wadsworth.org/research/scientific-resources/interactive-genomics. The position of the cleaved codons is given as codon position per total number of codons of the transcript. The cleaved codon sequence is indicated as "motif" and the bar slash shows where cleavage occurs. The position of the first nucleotide after cleavage in *M. smegmatis* genome is given in the "genomic position" column. The number of total detected cleaved RNA molecules (cleavages) for each transcript and the number of replicates in which such cleavages were identified (replicates) are shown. The presence (+) or absence (-) of homologous transcript (transcript) and conserved sequence motif (cleavage site) in *M. tuberculosis* (Mtb) is also shown. Essentiality of the identified genes in *M. smegmatis* (Msmeg; DOI: 10.1038/s41598-019-47905-y) or in *M. tuberculosis* (Mtb; doi: 10.1128/mBio.02133-16) is depicted a (+) essential or (-) non-essential. Functional annotation of conserved genes is based on information available on the Universal Protein Knowledgebase (UniProtKB). **b** Logoplots generated following alignment of all HigB^{TAC} target sequences identified for the WT (n=794), the Δ*rnJ* mutant (n=1243) or both WT + Δ*rnJ* (n=2037). The *x* axis represents the 10 nucleotides upstream and downstream of the cleavage site that is located by the position between -1 and 1 (red arrow and dash line), and default label for the *y* axis is bits (https://weblogo.berkeley.edu/logo.cgi). **c** Cleavages identified *in vivo* by nEMOTE for the inactive HigB^{TAC} [K95A] after 3 hours and for HigB2 and HigB3 after 24 hours of expression in *M. smegmatis* WT (blue bars on the left) and Δ*rnJ* (violet bars on the right) as shown in **Fig. 2a**. The name of the cleaved mRNA is given in line with each bar. The number of cleavages identified in each replicate for the WT (shade of blue; replicates I, II and III) and the Δ*rnJ* mutant (shade of violet; replicates I, II and III) is shown within each bar using the indicated color code. Bar height represents the number of independently observed cleavage events for each unique target site.

Supplementary Fig. 3

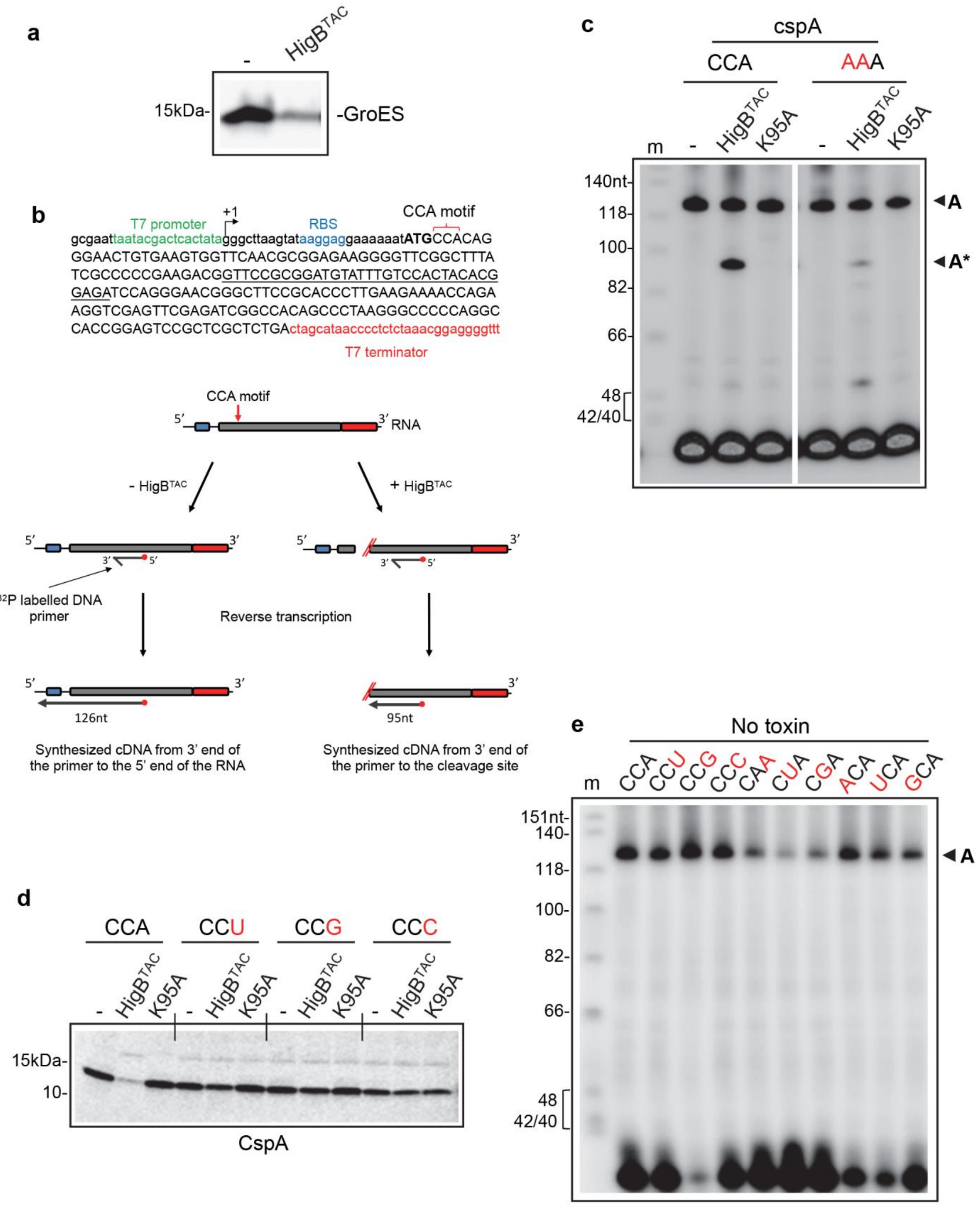

Supplementary Fig. 3: *Mtb*-GroES synthesis inhibition and *cspA* cleavage *in vitro*. a Strep-tagged *M. tuberculosis* GroES was expressed in a cell-free translation system with or without HigB^{TAC}. Reactions were performed for 1 h 30 min at 37 °C. After translation,

samples were separated on SDS–PAGE and visualized by western blot using an anti-Streptag II antibody. **b** Schematic representation of the primer extension experiment using cspA as template.. The nucleotide sequence of *cspA* is shown with the initiation codon in bold. The T7 promoter and terminator sequences are shown in green and red, respectively, and the ribosome binding site in blue. The labeled reverse DNA primer used for reverse transcription is underlined. Cleavage of the *cspA* mRNA in the CCA motif will lead to the synthesis of a 95nt cDNA. No cleavage will lead to the synthesis of a 126nt cDNA. **c** *cspA* cleavage by HigB^{TAC} wild-type or its non-toxic K95A derivative. *cspA* wild-type (CCA) or AAA mutant (AAA replaces CCA of Pro 2 codon) were independently expressed in a cell-free translation system for 2 h with or without HigB^{TAC} (1.5 µM). RNA were extracted and subjected to a primer extension with [^{32}P]-labeled *cspA* primer. Labeled cDNAs were separated on denaturing urea-polyacrylamide gel and revealed by autoradiography. Arrows show the uncleaved (A, 126 nt) and cleaved (A*, 95 nt) *cspA*. (m) stands for molecular ladder. Representative results of three independent experiments are shown. **d** Mutations in the CCA codon prevent inhibition of synthesis of *cspA* by HigB^{TAC} *in vitro*. *cspA* wild type (CCA) and its mutant derivatives (mutations depicted in red) were independently expressed in a cell-free translation system with or without 1.5 µM of HigB^{TAC} (or HigB^{TAC} [K95A] inactive mutant as control) and analyzed as described in **Fig. 3a**. Representative results of triplicate experiments are shown. **e** Control without toxin for primer extension experiments shown in **Fig. 3d**. A representative gel of two independent experiments is shown.

Supplementary Fig.4

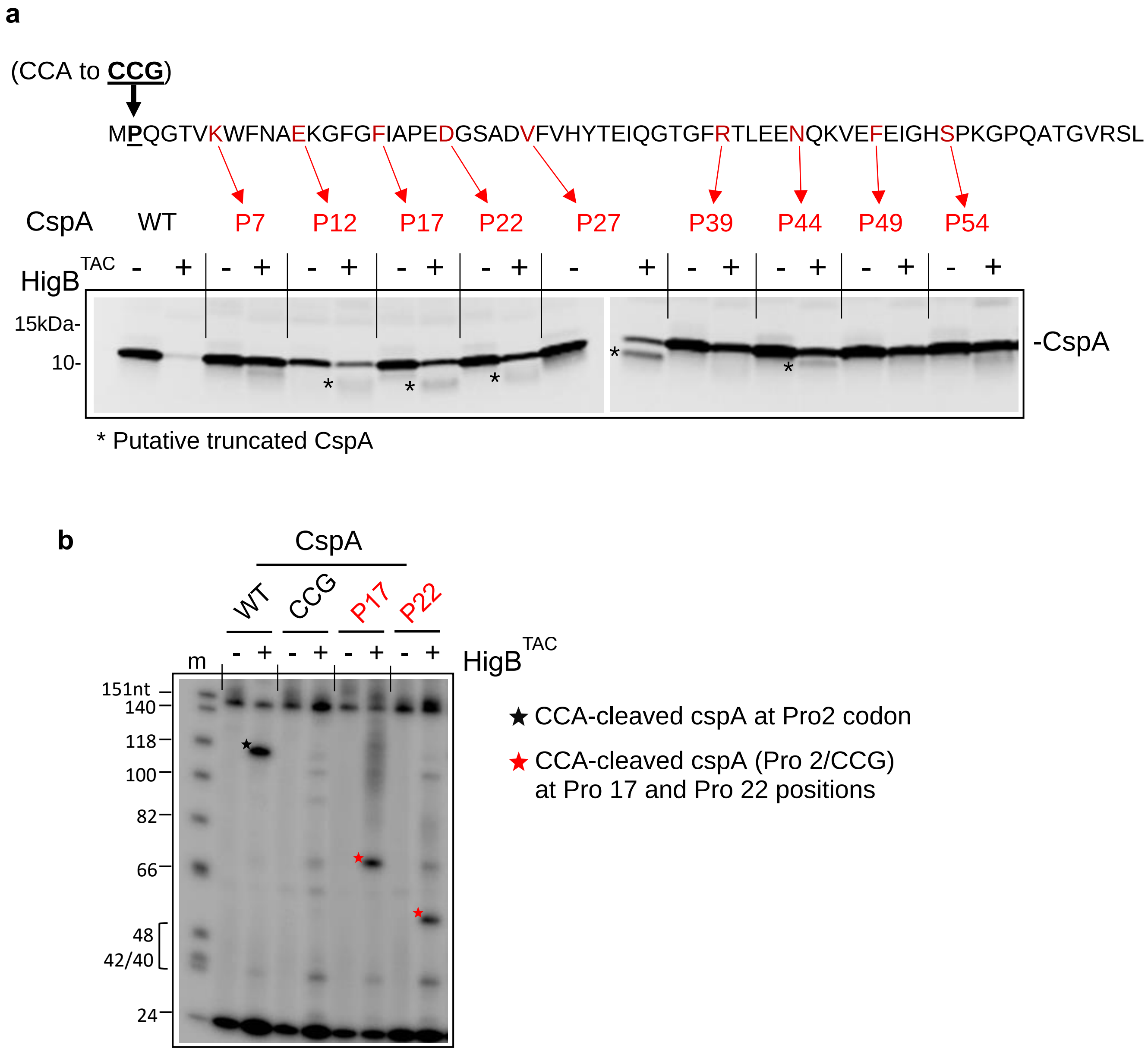

Supplementary Fig. 4: HigB^{TAC} can cleave along the mRNA during translation. a Inhibition of CspA synthesis with displaced Pro CCA codons along the *cspA* transcript. The amino acid sequence of CspA is shown on top, with each position independently substituted by a Pro (CCA codon) depicted in red. In these cases, the native Pro CCA codon is replaced by Pro CCG, which is not detectably cleaved by HigB^{TAC} (**Fig. 3c and d**). The asterisk indicates the putative truncated CspA. CspA WT, and its derivatives (P7 to P54) were independently expressed in a cell-free translation system with or without HigB^{TAC} (1.5 µM). CspA translation products were labelled with [^{35}S]methionine and reactions were performed for 1 h 30 min at 37 °C. After translation, samples were separated on SDS–PAGE and visualized by phosphorimager. **b** Cleavage of *cspA* and its P17 and P22 derivatives. *cspA*

(WT), P17 and P22 were independently expressed in a cell-free translation system for 2 h with or without $HigB^{TAC}$ (1.5 µM). RNAs were extracted and subjected to a primer extension with [^{32}P]-labeled *cspA* primer. The obtained labeled cDNAs were separated on denaturing urea-polyacrylamide gel and revealed by autoradiography. The black star shows the expectedly cleaved *cspA* wild-type product and red stars shows the shorter *cspA* cleavage products obtained when cleavage occurs at the newly positioned CCA codons (P17 at 72 nt and P22 at 57 nt). Representative results of three independent experiments are shown.

Supplementary Fig.5

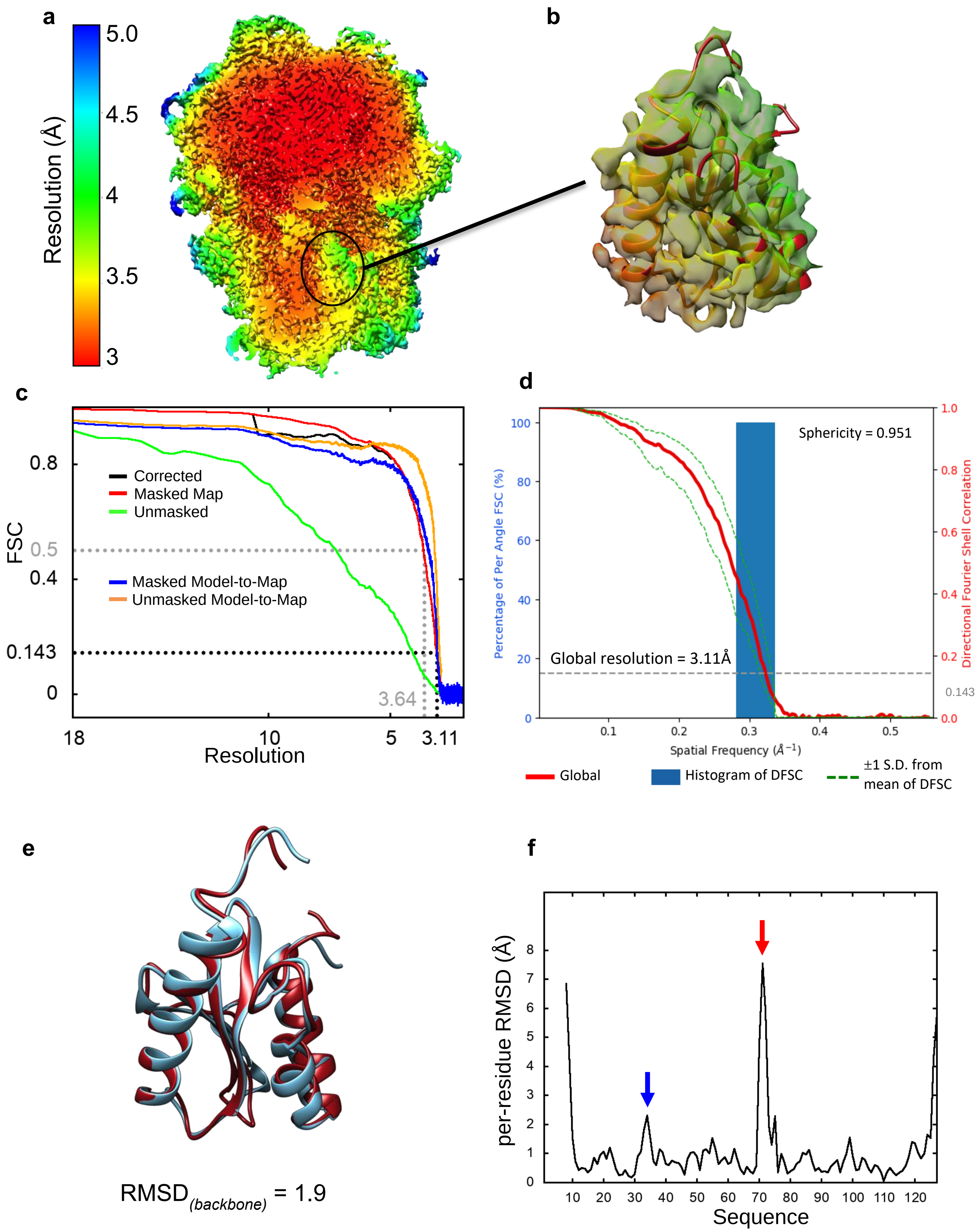

Supplementary Fig. 5: Evaluation of the quality of the map and models. a Sliced view of the consensus-sharpened density map colored according to the local resolutions computed

with Resmap (see color key). **b** Same, but a close-up to show the local resolution of HigB^{TAC} [K95A]. **c** Fourier shell correlation (FSC) curves calculated between the unmasked, masked, or solvent-corrected half-maps, and between the atomic model and the unmasked or masked consensus-sharpened maps. Dashed lines indicate *FSC* = 0.143 (black) and 0.5 (grey). The corresponding resolutions calculated for the solvent-corrected half-maps are also indicated. **d** Directional 3DFFSC plot showing the global half-map FSC (solid red line), the spread of directional resolution values defined by ±1σ from the mean (dotted green lines, left axis) and a histogram of 100 such values evenly sampled over the 3D FSC (blue bars, right axis). **e** Superimposition of the atomic coordinates of HigB^{TAC} [K95A] alone (our crystal structure, blue) and observed on a translating ribosome (our cryo-EM model, red). The root-mean-square deviation (RMSD) calculated for the backbone atoms is indicated. **f** Average per-residue RMSD calculated between the two HigB^{TAC} [K95A] structures. With the exception of the extremities, the two structures mostly differ in the region between the first and second alpha helices (blue arrow), and between the second and third beta strand (red arrow).

Supplementary Fig. 6

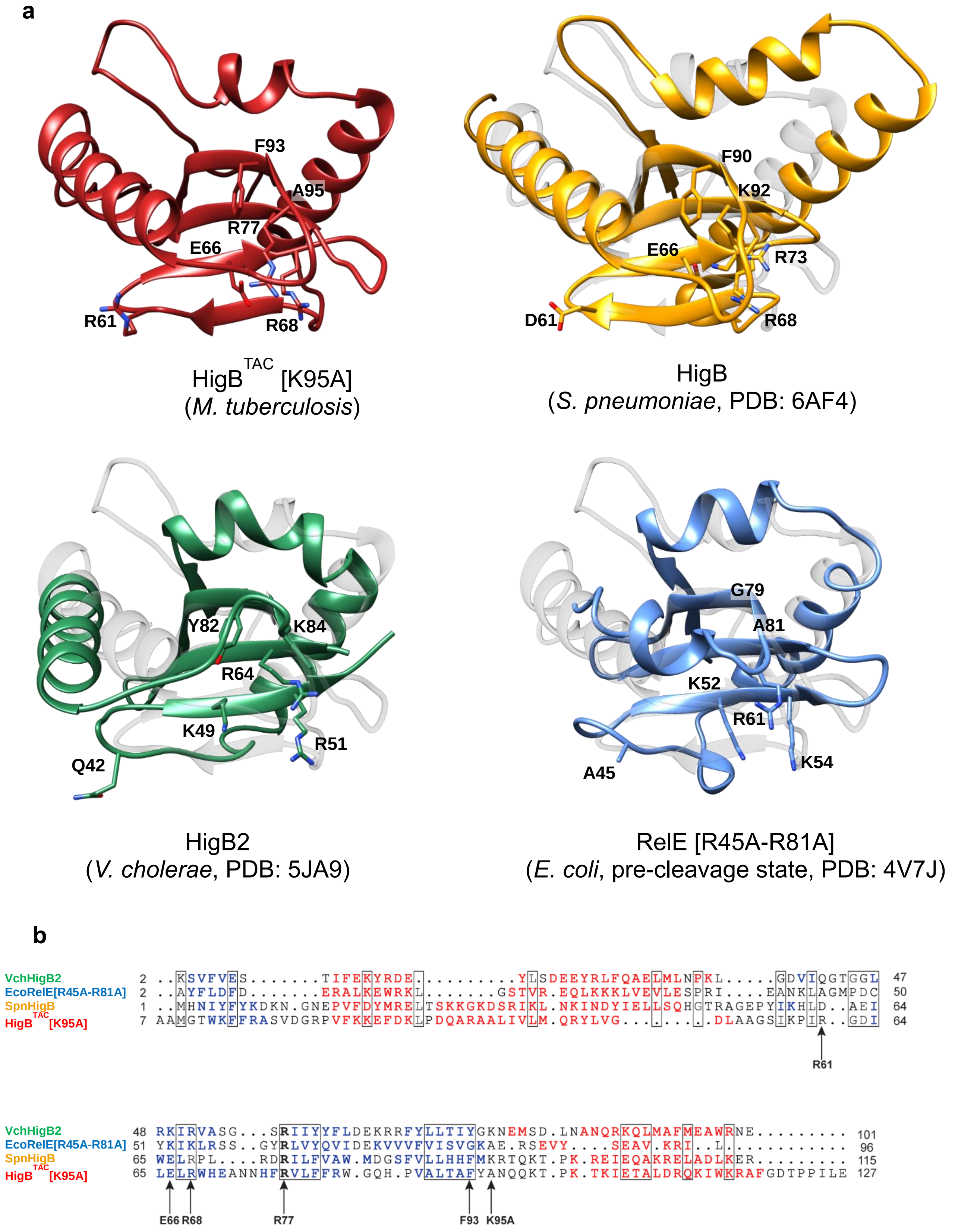

Supplementary Fig. 6: Comparison of the catalytic sites of ribosome-dependent toxins. a Structure showing the proposed catalytic-site residues in HigB^{TAC} [K95A], *S. pneumoniae*

HigB (PDB 6AF4), *V. cholera* HigB2 (PDB 5JA9), and in *E. coli* RelE [R45A-R81A] (PDB 4V7J). All the structure were aligned on HigB^{TAC} [K95A] using UCSF chimera and, for comparison, the structure of HigB^{TAC} [K95A] (transparent white) is superimposed to the other toxins. **b** Structure-derived protein sequence alignments obtained using PROMALS3D and submitted to ESPript 3.0 for amino acid similarity analysis. Conserved residues are indicated in bold. Residues with high similarity are shown in a black frame. Residues in red and blue correspond to α-helix and β-strand, respectively. Residues that are part of the catalytic center are indicated with arrows.

Supplementary Fig. 7

a

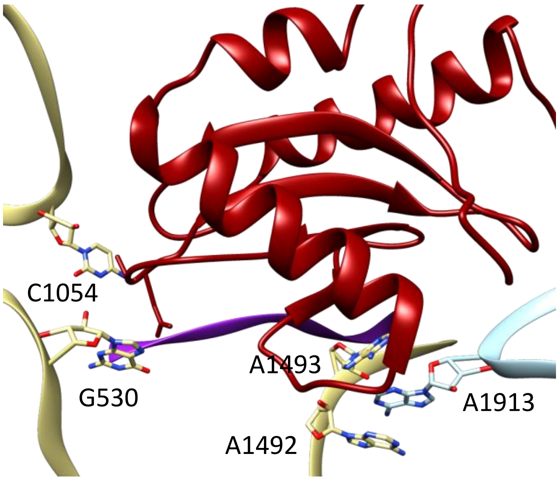

HigB^{TAC} [K95A]
(*M. tuberculosis*)

b

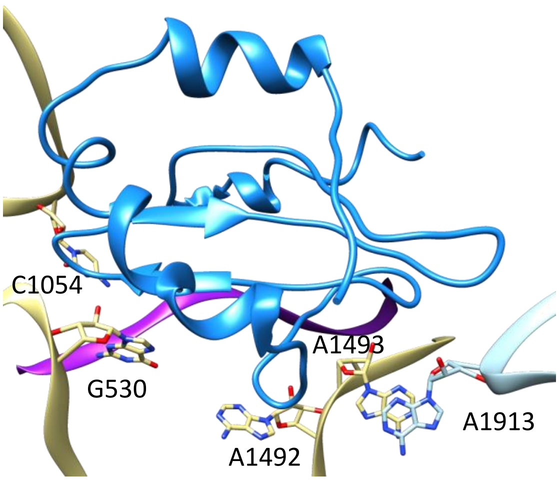

RelE [R45A-R81A]
(*E. coli*, pre-cleavage state)

c

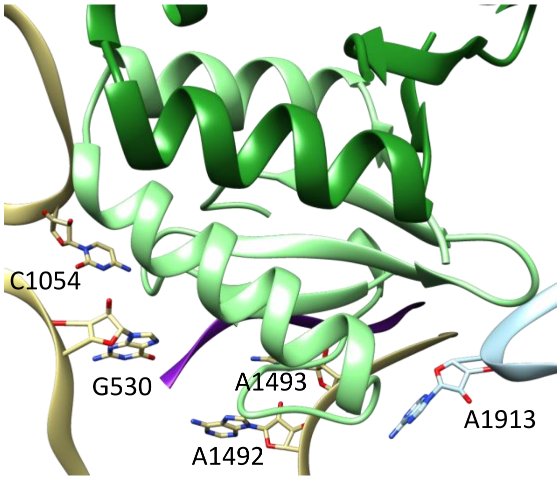

YoeB dimer
(*E. coli*, pre-cleavage state)

d

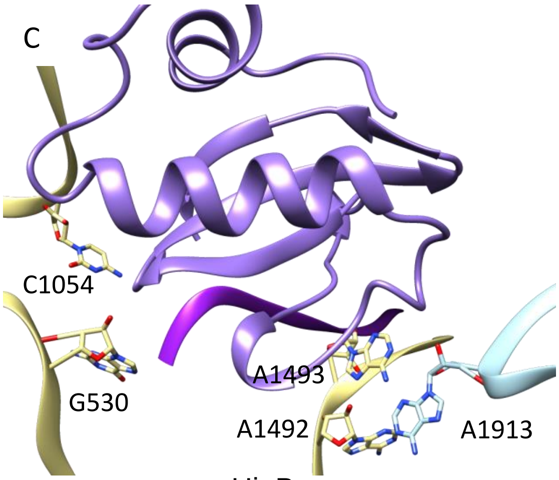

HigB
(*P. Vulgaris*)

e

Contacts		HigB^{TAC} [K95A]	RelE [R45A-R81A]	YoeB dimer	HigB
fMet-tRNA^{fMet}		C29-G30, A36-U37			A35-U36
CspA mRNA		C30-C33	(A2M)20-A22	G18-A22	G18-(A2M)21
23S rRNA	H69	A1912	A1913	A1913-U1915	
16S rRNA	h18	G517-C518, G530-U531	U516-C519, G530-A532	G517-A520, G530-U531	G517, C519, G530-U531
	h30-31	G953-A959, U965-m^{2}G966	U955, U957, A959-U960	U955-A959	G954, U957, U960-U961
	h34	U1052-C1054	G1050-A1055, G1198, C1208	C1054	G1050-C1054, C1208-C1209
	h32				C1214
	h44	A1492-A1493	A1492-G1494	A1492-U1495	A1493
uS13		R117			
uS12			S50	T44, P48, S50-L52	T44, S50, A51

Supplementary Fig. 7: Effect of toxins on the decoding site and summary of all the contacts made by toxins with the ribosome, their target mRNAs, and eventually with P-site tRNA. Contact of decoding site residues with **a** HigB^{TAC} [K95A], **b** *E. coli* RelE [R45A-R81A] (PDB 4V7J), **c** *E. coli* YoeB dimer (PDB 4V8X), and **d** *P. vulgaris* HigB (PDB 4ZSN). The four structures are aligned on the 16S rRNA using UCSF chimera. The mRNA is purple, the 16S rRNA is light yellow, the 23S rRNA is light blue, the ribosomal proteins uS12 and uS13 are gold and lime green, respectively. Nucleotides within 4 Å of the toxin are shown. **e** Table summarizing all the interactions found between known toxins with the ribosome, their target mRNAs, and eventually with P-site tRNA.

Supplementary Fig. 8

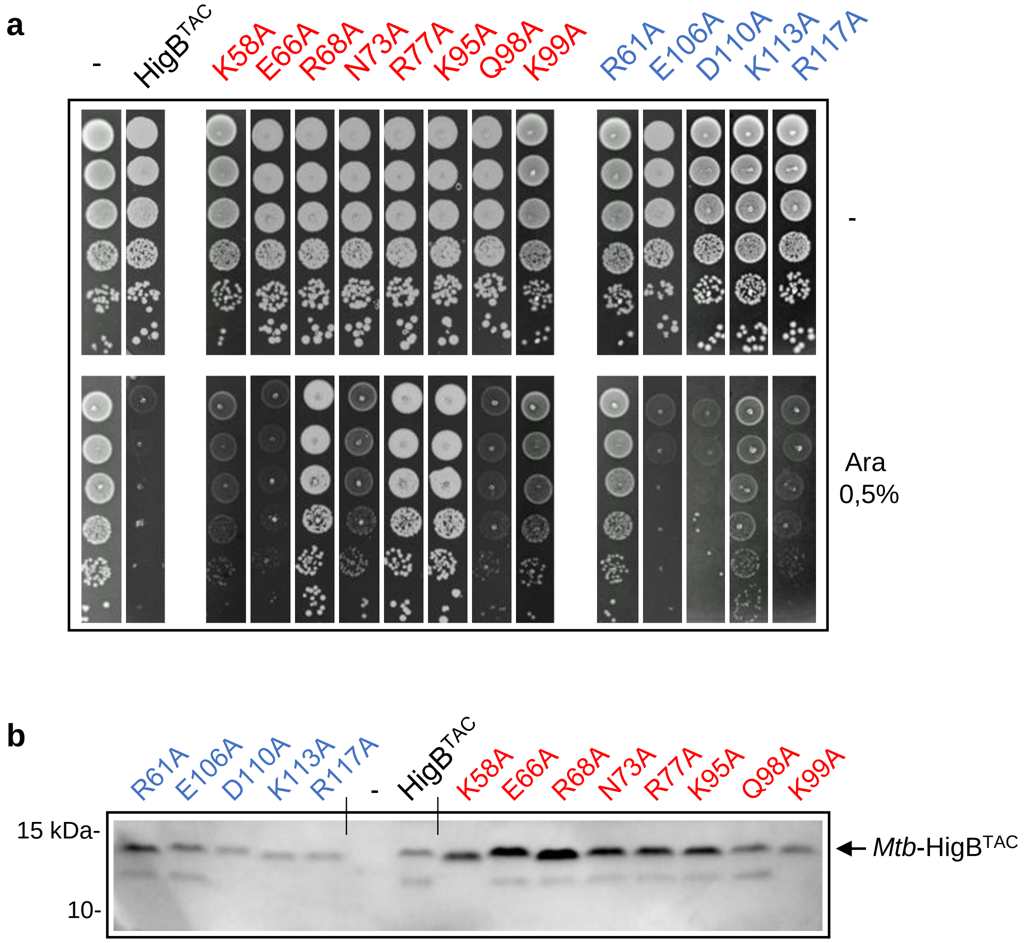

Supplementary Fig. 8: Toxicity of HigB^{TAC} derivatives in *E. coli.* a *E. coli* W3110 strain transformed with pMPMK6-vector, HigB^{TAC} wild-type or its mutant derivatives (Alanine substitution of residue K58, E66, R68, N73, R77, K95, Q98, K99, R61, E106, D110, K113 or R117) were serial diluted, spotted on LB kanamycin agar plates with or without arabinose (0.5%) inducer and incubated 1 day at 37°C. **b** Western blot showing HigB^{TAC} steady state expression level following a 3h induction in the presence of 0.5% arabinose. Representative results of two independent experiments are shown.

Supplementary Fig. 9

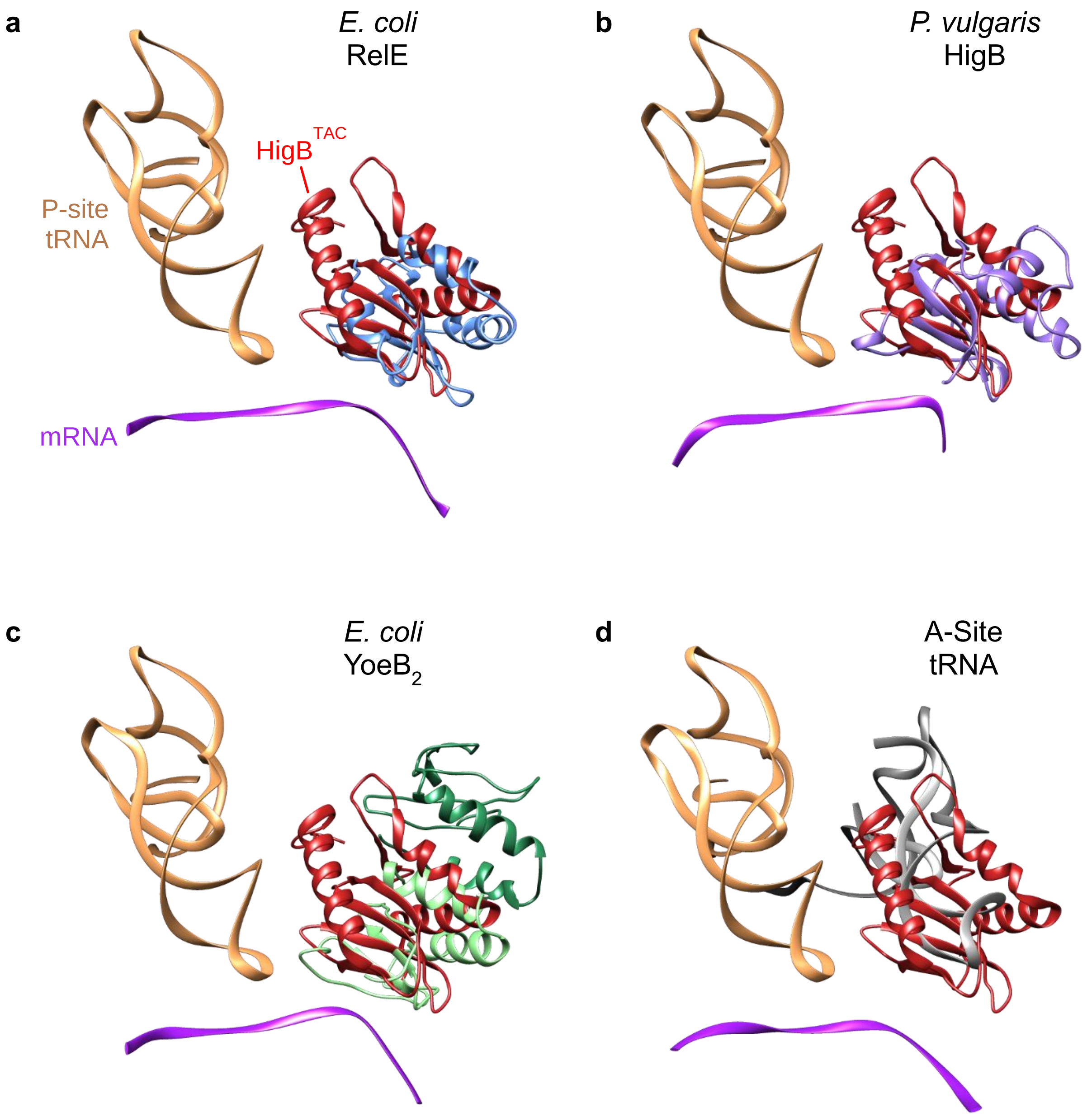

Supplementary Fig. 9: Structural comparison between HigB^{TAC}[K95A] and known toxins observed in pre-cleavage state with their respective mRNA targets and the P-site tRNA. a *E. coli* RelE[R45A_R81A] (PDB code 4V7J), **b** *P. vulgaris* HigB (PDB 4ZSN), **c** *E. coli* YoeB dimer (PDB 4V8X), and **d** a cognate tRNA observed in the A-site during canonical translation (PDB 7K00). The structures were all aligned, using UCSF chimera, on the P-site tRNA of the ribosome-associated TAC toxin. For clarity reason only the HigB^{TAC}[K95A] (in red) is shown superimposed to the other structures.

Supplementary Fig. 10

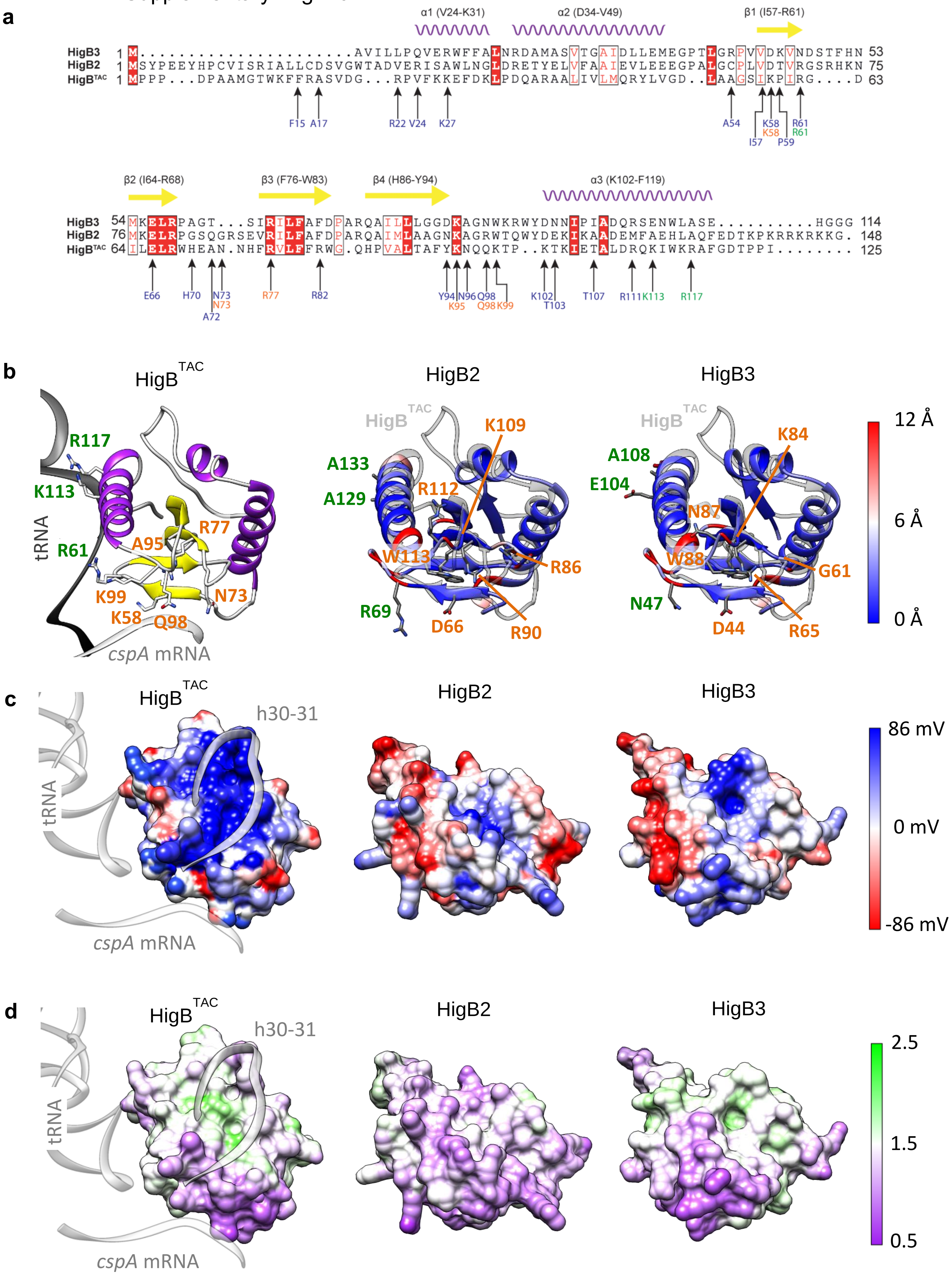

Supplementary Fig. 10: Comparison of *M. tuberculosis* HigB toxins. a Alignment of HigB toxins was performed using PROMALS3D and submit to ESPript 3.0 for amino acid similarity analysis. Conserved residues are shown in red background and blue frame. Residues with high similarity are shown in red and black frame. Symbols above sequence alignment correspond to the secondary structure of HigB^{TAC} with α-helix and β- sheet colored in purple and yellow respectively. Residues that are within 3Å of the 16S and 23S are shown in blue, and those within 3Å of fMet-tRNA^{fMet} in green. Residues located in the catalytic center and in contact with *cspA* mRNA are shown in orange. **b** Comparison between our cryo-EM structure of HigB^{TAC} [K95A] mutant and the AlphaFold[9] models of *M. tuberculosis* HigB2 and HigB3. The three proteins are aligned with STAMP[10]. HigB^{TAC} is colored based on its secondary structure (purple for the helices and yellow for the strands) and the position of the P-site tRNA, and CspA mRNA are indicated. HigB2 and HigB3 are colored based on the per-residue RMSD with HigB^{TAC} from blue (low RMSD) to red (High RMSD) and for comparison, the structure of HigB^{TAC} [K95A] (transparent white) is superimposed to the two other toxins. Residues located in the catalytic center or in contact with fMet-tRNA^{fMet} as well as their equivalent in HigB2 and HigB3 are shown. **c** Electrostatic surface potential calculated with Protein-sol[11]. The proteins are colour-coded from negatively charge (red) to positively charge (blue) and the range of potential given here is in the range of what is expected for electrostatic interactions at the protein surface. The position of the P-site tRNA, CspA mRNA, and the junction between 16S rRNA helices 30 and 31 are also indicated. **d** Same as **c** but for hydrophobicity. The proteins are color-coded by patch NPP ratio from polar (purple) to more hydrophobic (green).

Supplementary Fig. 11

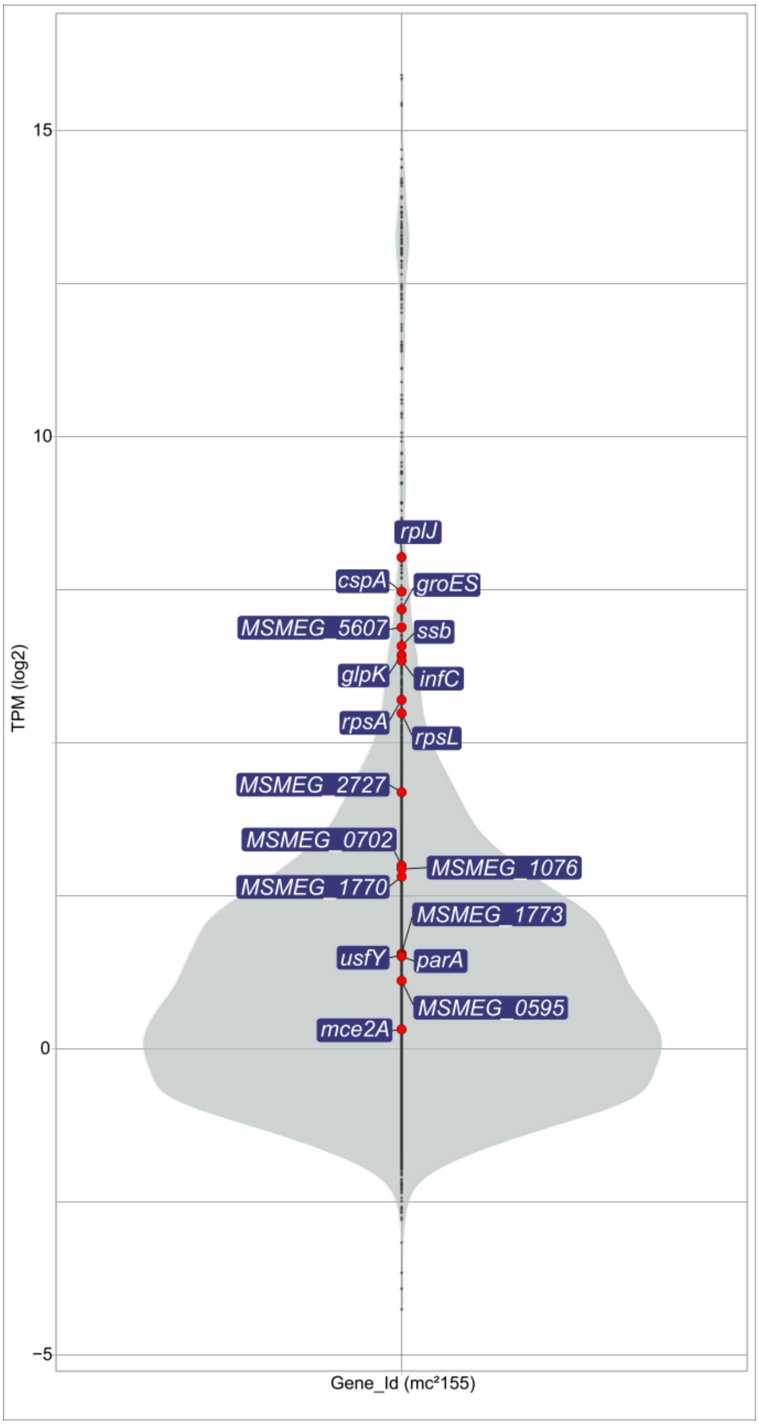

Supplementary Fig. 11: *M. smegmatis* mRNA expression. Violin plot showing the distribution of the intensity of expression in *M. smegmatis* obtained from available transcriptomic data. The x-axis represents the total genes of *M. smegmatis* with the transcripts cleaved by HigB^{TAC} highlighted with red dots and on the y-axis the representation of gene expression in transcripts per million (TPM). Violin Plot was generated with ggplot in RStudio.

Supplementary Fig. 12

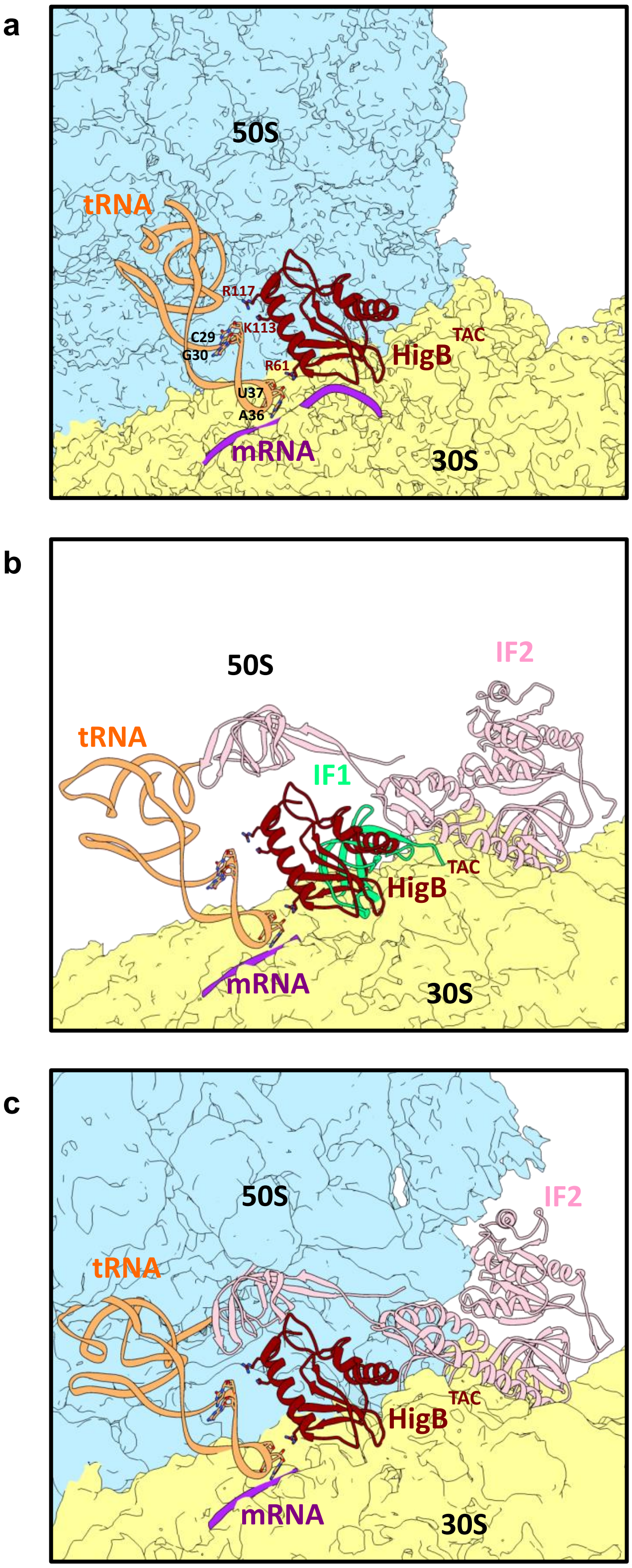

Supplementary Fig. 12: HigB^{TAC} potentially targets the late steps of the translation initiation. **a** Close-up view of the ribosome-associated HigB^{TAC} with its native *cspA* substrate. **b** Structural comparison between the 30S initiation complex (PDB 6O7K, EMD-0643) and the ribosome-associated HigB^{TAC} shows that IF1 and the toxin are mutually exclusive. **c** Structural comparison between the 70S initiation complex (PDB 6O9K, EMD-0662) and the ribosome-associated HigB^{TAC}. The presence of IF2 stabilizes the ribosome in a semi-rotated state and the fMet-tRNA^{fMet} in its P/I configuration. This prevents the tRNA-EF-Tu complex to bind the ribosome but does not impede HigB^{TAC} to access the A-site and interact with the P-Site tRNA. The structures are aligned on the 16S rRNA. HigB^{TAC} is red, the mRNA is purple, the fMet-tRNA^{fMet} is orange, IF1 is green, IF2 is pink and the electron density maps of the 50S and 30S ribosomal subunits are blue and yellow respectively. HigB^{TAC} residues R61, K113 and R117 and the fMet-tRNA^{fMet} nucleotides C29, G30, A36, and U37 are also shown to highlight that HigB^{TAC} can contact the fMet-tRNA^{fMet}, even if it is in the P/I configuration.

Supplementary Fig. 13

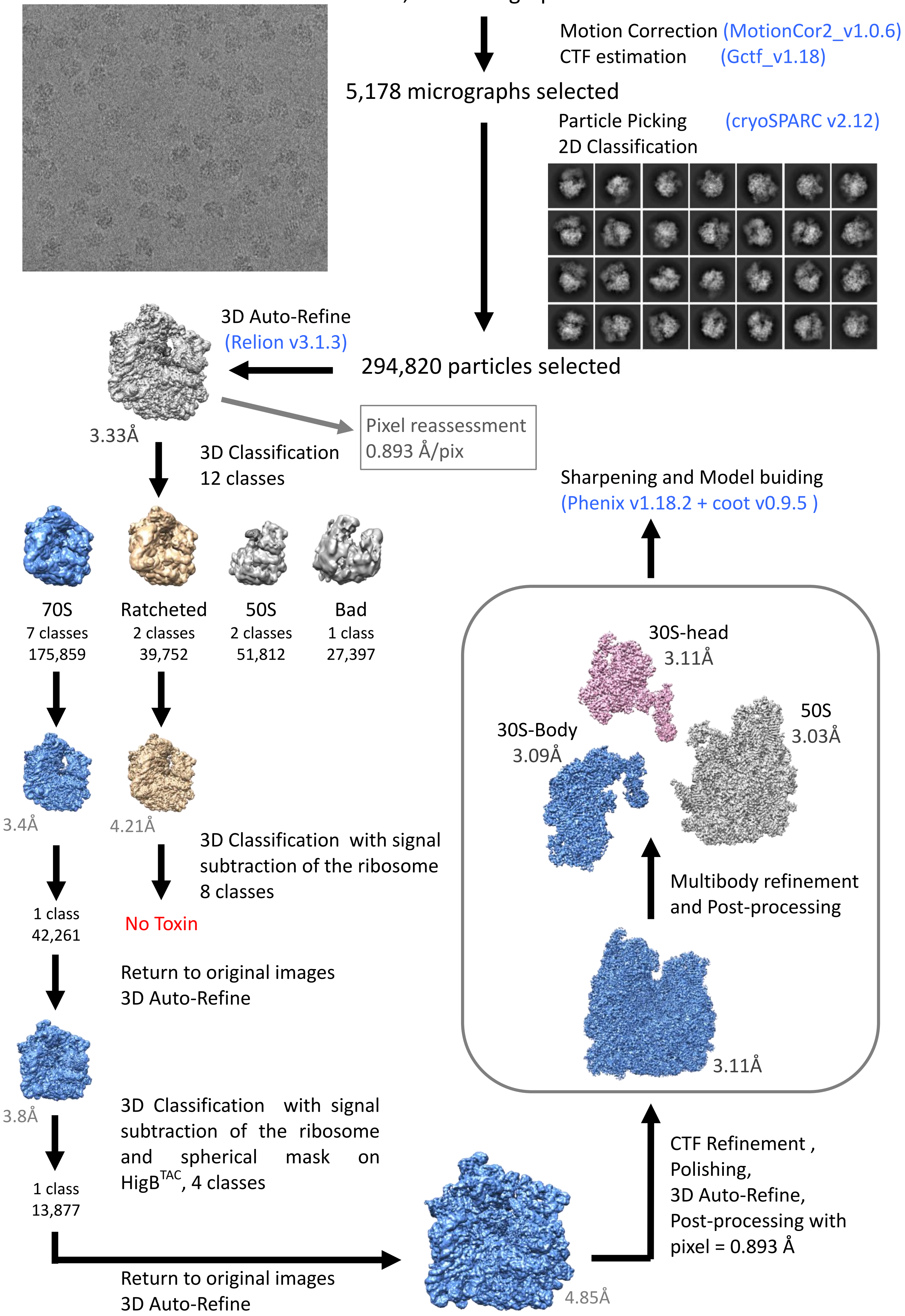

Supplementary Fig. 13: Schematic representation of the cryo-EM single-particle reconstruction workflow.

Supplementary Table 1.

Crystallographic data collection and refinement statistics ([a]Values in brackets are for the highest-resolution shell and [b]Asymmetric unit)

**Data set**	*Mtb*-HigB1^{K95A}
**PDB code**	7AWK
**Data collection**	
Beamline	ESRF MASSIF-3
Space group	$P2_12_12$
Unit cell *a*, *b*, *c* (Å)<br>[α, β, γ (°)] for *P*1	43.98, 82.05, 38.05
Resolution range (Å)[a]	41.03 – 1.91<br>(1.95 – 1.91)
No. of unique reflections	11,202 (739)
Completeness (%)	98.9 (92.7)
Redundancy	6.7 (6.4)
$<I/\sigma(I)>$	9.2 (1.3)
R_{meas} (%)	12.0 (131.8)
CC1/2	0.991 (0.602)
Wilson B factor (Å²)	36.6
**Refinement**	
No. of reflections<br>(work / test)	10,632 (560)
R_{work} / R_{free}	0.212 / 0.273
No. of molecules/AU[b]	1
No. of non-hydrogen atoms	1,049
Protein	984
Ligands	0
Ions	0
Water molecules	65
RMS deviations	
Bond lengths (Å)	0.013
Bond angles (°)	1.833
Ramachandran plot (%)	
Most favored	97.5
Allowed / disallowed	2.5 / 0
Average B-factors (Å^2)	40.6
Protein	40.4
Solvent	44.5

Supplementary Table 2.

Cryo-EM data collection and refinement statistics.

**EMDB code**	(EMD-12261)
**PDB code**	(PDB 7NBU)
**Data collection and processing**	
Magnification	105k
Voltage (kV)	200
Electron exposure (e–/Å^2)	35
Defocus range (μm)	-0.4 to -2
Pixel size (Å)	0.9291 (fitted to 0.893)
Symmetry imposed	None
Micrographs collected (no.)	6,381
Micrographs used (no.)	6,381
Initial particle images (no.)	294,820
Final particle images (no.)	13,877
Map resolution (Å)	
FSC threshold : 0.143	3.11
FSC threshold : 0.5	3.64
Map resolution range (Å)	2.9-7
**Refinement**	
Initial models used (PDB code)	7K00 and 7AWK
Model resolution (Å)	
FSC threshold: 0.143	2.9
Map sharpening	
B factor (Å^2)	26.58
Model composition	
Non-hydrogen atoms	144,943
Protein residues	5,719
Nucleotide	4,648
Ligands	317
B factors mean (Å^2)	
Protein	87.67
Nucleotide	97.17
Ligand	61.85
RMS deviations	
Bond lengths (Å)	0.007
Bond angles (°)	0.772
Validation	
MolProbity score	1.93
Clashscore	10.28
Poor rotamers (%)	0.09
Ramachandran plot	
Favored (%)	94.03
Allowed (%)	5.78
Disallowed (%)	0.20

Supplementary Table 3. Primers list

Primer name	Sequence (5’-3’)
**Toxins cloning in pGMC**	
pGMC_Infusion_Fw	CAACTTTATTATACATAGTTGATAATTC
pGMC_Infusion_Rv	GGGCAGCCTGTCTTCCTC
HigB1_pGMC_Fw	GAAGACAGGCTGCCCATGCCGCCCCCTGATCCAGCCGCCATG
HigB1_pGMC_Rv	TGTATAATAAAGTTGTTAGATCGGTGGGGTGTCGCCGAAG
HigB2_pGMC_Fw	GAAGACAGGCTGCCCATGTCTTACCCGGAGGAATATCACCCTTG
HigB2_pGMC_Rv	TGTATAATAAAGTTGTTACCCTTTCTTGCGCTTGCGCCTC
HigB3_pGMC_Fw	GAAGACAGGCTGCCCATGGCCGTGATCCTGCTCCCGCAG
HigB3_pGMC_Rv	TGTATAATAAAGTTGTTATCCACCTCCGTGCTCGCTC
HigB1_R68A_pGMC_Fw	GACATTCTGGAGTTGGCCTGGCATGAGGCGAAC
HigB1_R68A_pGMC_Rv	GTTCGCCTCATGCCAGGCCAACTCCAGAATGTC
HigB1_R77A_pGMC_Fw	GCGAACAACCACTTCGCCGTACTGTTCTTCCGC
HigB1_R77A_pGMC_Rv	GCGGAAGAACAGTACGGCGAAGTGGTTGTTCGC
HigB1_K95A_pGMC_Fw	CTGACAGCGTTCTACGCCAACCAGCAGAAGACTC
HigB1_K95A_pGMC_Rv	GAGTCTTCTGCTGGTTGGCGTAGAACGCTGTCAG
**Toxins cloning in pLAM**	
pLAM_Infusion_Fw	GAATTCGAAGCTTATCGATG
pLAM_Infusion_Rv	CATATGTGGACTCCCTTTC
HigB1_pLAM_Fw	GGGAGTCCACATATGCCGCCCCCTGATCCAGCCGCCATG
HigB1_pLAM_Rv	ATAAGCTTCGAATTCTCAGATCGGTGGGGTGTCGCCGAAG
HigB1_K95A_pLAM_Fw	CTGACAGCGTTCTACGCCAACCAGCAGAAGACTC
HigB1_K95A_pLAM_Rv	GAGTCTTCTGCTGGTTGGCGTAGAACGCTGTCAG
HigB2_pLAM_Fw	GGGAGTCCACATATGTCTTACCCGGAGGAATATCACCCTTG
HigB2_pLAM_Rv	ATAAGCTTCGAATTCTTACCCTTTCTTGCGCTTGC
HigB3_pLAM_Fw	GGGAGTCCACATATGGCCGTGATCCTGCTCCCGCAG
HigB3_pLAM_Rv	ATAAGCTTCGAATTCTCATCCACCTCCGTGCTCGC
**Toxins cloning in pBAD33**	
pBAD33_Infusion_Fw	GGCTGTTTTGGCGGATGAGAGAAGATTTTCAGC
pBAD33_Infusion_Rv	CGAGACAACCTCCTGCTAGCCCAAAAAAACGGGTATGGA
HigB2_pBAD33_Fw	CAGGAGGTTGTCTCGATGtcttacccggaggaata
HigB2_pBAD33_Rv	TCCGCCAAAACAGCCTTACCCTTTCTTGCGCTTG
HigB3_pBAD33_Fw	CAGGAGGTTGTCTCGATGGCCGTGATCCTGCTCCCG
HigB3_pBAD33_Rv	TCCGCCAAAACAGCCTCATCCACCTCCGTGCTCGCTCGCC
**nEMOTE primers**	
Rp8	5'**Bio**CGGCACCAACCGAGGVVVVVVVACAGA V (A or C or G)
D6A	CTCTTTCCCTACACGACGCTCTTCCGATCTNTACACGGCACCAACCGAGG
D6B	CTCTTTCCCTACACGACGCTCTTCCGATCTNGTATCGGCACCAACCGAGG
D6C	CTCTTTCCCTACACGACGCTCTTCCGATCTNCGTCCGGCACCAACCGAGG
D6D	CTCTTTCCCTACACGACGCTCTTCCGATCTNAAGTCGGCACCAACCGAGG
D6E	CTCTTTCCCTACACGACGCTCTTCCGATCTNACACCGGCACCAACCGAGG

D6F	CTCTTTCCCTACACGACGCTCTTCCGATCTNGGTACGGCACCAACCGAGG
D6H	CTCTTTCCCTACACGACGCTCTTCCGATCTNTCGGCGGCACCAACCGAGG
D6I	CTCTTTCCCTACACGACGCTCTTCCGATCTNCAAGCGGCACCAACCGAGG
D6J	CTCTTTCCCTACACGACGCTCTTCCGATCTNTTGACGGCACCAACCGAGG
D6K	CTCTTTCCCTACACGACGCTCTTCCGATCTNGCTGCGGCACCAACCGAGG
D6L	CTCTTTCCCTACACGACGCTCTTCCGATCTNCCGACGGCACCAACCGAGG
D6M	CTCTTTCCCTACACGACGCTCTTCCGATCTNCTCGCGGCACCAACCGAGG
D6N	CTCTTTCCCTACACGACGCTCTTCCGATCTNAGGACGGCACCAACCGAGG
D6O	CTCTTTCCCTACACGACGCTCTTCCGATCTNATTGCGGCACCAACCGAGG
D6P	CTCTTTCCCTACACGACGCTCTTCCGATCTNGACGCGGCACCAACCGAGG
D6Q	CTCTTTCCCTACACGACGCTCTTCCGATCTNTGTTCGGCACCAACCGAGG
DRNA	GGCATTCCTGCTGAACCGCTCTTCCGATCTNNNNNNNNNA
Adaptator A	AATGATACGGCGACCACCGAGATCTACACTCTTTCCCTACACGACG
Adaptator B	CAAGCAGAAGACGGCATACGAGATCGGTCTCGGCATTCCTGCTGAACCGC

Toxin cloning in pET vector

pET20b_Infusion_Fw	ATGTATATCTCCTTCTTAAAG
pET20b_Infusion_Rv	CACCACCACCACCACCACTG
higB2_infusion_20b_for	GAAGGAGATATACATATGtcttacccggaggaatatc
higB2_infusion_20b_rev	GTGGTGGTGGTGGTGCCCTTTCTTGCGCTTGCGCC
HigB3_ infusion_20b_for	GAAGGAGATATACATaTGGCCGTGATCCTGCTCCCG
HigB3_ infusion_20b_rev	GTGGTGGTGGTGGTGTCCACCTCCGTGCTCGCTCGCC
HigB1_pET20b_NdeI_Fw	Texier *et al* 2021
HigB1_pET20b_XhoI_Rv	Texier *et al* 2021
HigB1opt_NdeI_Fw	ATACATATGCCGCCGCCGGACCCGGCAG
HigB1opt_BamHI_Rv	TTTGGATCCTTACTCGAGGATCGGCGGGGTATCACC

PURE with cspA

pSE_Infusion_Fw	CCGGTTGTTAACGTTAGCCGG
pSE_Infusion_Rv	GCTGGAATTCTCCTGTGTGAAATTG
CspA_IF_Fw	CAGGAGAATTCCAGCATGCCACAGGGAACTGTGAAG
CspA_IF_Rv	AACGTTAACAACCGGTCATTTTTCGAACTGCGGGTGGCTCCAGCTACCCCCGAGCG AGCGGACTCCGGTGGCC
CspA_CCA_Fw	GCGAATTAATACGACTCACTATAGGGCTTAAGTATAAGGAGGAAAAAATATGCCAC AGGGAACTGTGAAG
CspA_term_Rv	AAACCCCTCCGTTTAGAGAGGGGTTATGCTAGTCAGAGGGAGCGGACTCCGGTGG CCTG
CspA_CCT_Fw	GCGAATTAATACGACTCACTATAGGGCTTAAGTATAAGGAGGAAAAAATATGCCTC AGGGAACTGTGAAG
CspA_CCG_Fw	GCGAATTAATACGACTCACTATAGGGCTTAAGTATAAGGAGGAAAAAATATGCCGC AGGGAACTGTGAAG
CspA_CCC_Fw	GCGAATTAATACGACTCACTATAGGGCTTAAGTATAAGGAGGAAAAAATATGCCCC AGGGAACTGTGAAG

CspA_CAA_Fw	GCGAATTAATACGACTCACTATAGGGCTTAAGTATAAGGAGGAAAAAATATGCAACAGGGAACTGTGAAG
CspA_CTA_Fw	GCGAATTAATACGACTCACTATAGGGCTTAAGTATAAGGAGGAAAAAATATGCTACAGGGAACTGTGAAG
CspA_CGA_Fw	GCGAATTAATACGACTCACTATAGGGCTTAAGTATAAGGAGGAAAAAATATGCGACAGGGAACTGTGAAG
CspA_ACA_Fw	GCGAATTAATACGACTCACTATAGGGCTTAAGTATAAGGAGGAAAAAATATGACACAGGGAACTGTGAAG
CspA_TCA_Fw	GCGAATTAATACGACTCACTATAGGGCTTAAGTATAAGGAGGAAAAAATATGTCACAGGGAACTGTGAAG
CspA_GCA_Fw	GCGAATTAATACGACTCACTATAGGGCTTAAGTATAAGGAGGAAAAAATATGGCACAGGGAACTGTGAAG
CspA_codon 7_Fw	GCGAATTAATACGACTCACTATAGGGCTTAAGTATAAGGAGGAAAAAATATGCCGCAGGGAACTGTGCCA
CspA_codon 12_Fw	AAGTGGTTCAACGCGCCAAAGGGGTTCGGCTTT
CspA_codon 12_Rv	AAAGCCGAACCCCTTTGGCGCGTTGAACCACTT
CspA_codon 17_Fw	GAGAAGGGGTTCGGCCCAATCGCCCCCGAAGAC
CspA_codon 17_Rv	GTCTTCGGGGGCGATTGGGCCGAACCCCTTCTC
CspA_codon 22_Fw	TTTATCGCCCCCGAACCAGGTTCCGCGGATGTA
CspA_codon 22_Rv	TACATCCGCGGAACCTGGTTCGGGGGCGATAAA
CspA_codon 27_Fw	GACGGTTCCGCGGATCCATTTGTCCACTACACG
CspA_codon 27_Rv	CGTGTAGTGGACAAATGGATCCGCGGAACCGTC
CspA_codon 39_Fw	ATCCAGGGAACGGGCCCACGCACCCTTGAAGAA
CspA_codon 39_Rv	TTCTTCAAGGGTGCGTGGGCCCGTTCCCTGGAT
CspA_codon 44_Fw	TTCCGCACCCTTGAACCAAACCAGAAGGTCGAG
CspA_codon 44_Rv	CTCGACCTTCTGGTTTGGTTCAAGGGTGCGGAA
CspA_codon 49_Fw	GAAAACCAGAAGGTCCCATTCGAGATCGGCCAC
CspA_codon 49_Rv	GTGGCCGATCTCGAATGGGACCTTCTGGTTTTC
CspA_codon 54_Fw	GAGTTCGAGATCGGCCCAAGCCCTAAGGGCCCC
CspA_codon 54_Rv	GGGGCCCTTAGGGCTTGGGCCGATCTCGAACTC
OOF1_Fw	GCGAATTAATACGACTCACTATAGGGCTTAAGTATAAGGAGGAAAAAATATGACCAAGGGAACTGTGAAG
OOF2_Fw	GCGAATTAATACGACTCACTATAGGGCTTAAGTATAAGGAGGAAAAAATATGCACCAGGGAACTGTGAAG
CspA_AAA_Fw	GCGAATTAATACGACTCACTATAGGGCTTAAGTATAAGGAGGAAAAAATATGAAACAGGGAACTGTGAAG
RT_CspA_P32_PAGE_1	TCTCCGTGTAGTGGACAAATACATCCGCGGAAC
RT_CspA_P32_PAGE_2	GTGCGGAAGCCCGTTCCCTGGATCTC

PURE with gfp

FPUREGfpT7	(Bordes et al., 2016)[5]
RPUREGfpT7	(Bordes et al., 2016)[5]

PURE with groES

GroES_IF_Fw	CAGGAGAATTCCAGCATGGCGAAGGTGAACATCAAG
GroES-strep_IF_Rv	AACGTTAACAACCGGTCATTTTTCGAACTGCGGGTGGCTCCAGCTACCCCCCTTGGAAACGACGGCCAG

GroES_T7_Fw	GCGAATTAATACGACTCACTATAGGGCTTAAGTATAAGGAGGAAAAAATATGGCG AAGGTGAACATCAAGCCACTCGAGGAC
GroES-strepterm_Rv	AAACCCCTCCGTTTAGAGAGGGGTTATGCTAGTCATTTTTCGAACTGCGGGTGGC

Supplementary Data 1. Data file (separate excel data file): nEMOTE data

Supplementary References

1. Mayer, M. P. A new set of useful cloning and expression vectors derived from pBlueScript. *Gene* **163**, 41–46 (1995).
2. Texier, P. *et al.* ClpXP-mediated Degradation of the TAC Antitoxin is Neutralized by the SecB-like Chaperone in Mycobacterium tuberculosis. *Journal of Molecular Biology* **433**, 166815 (2021).
3. Guzman, L. M., Belin, D., Carson, M. J. & Beckwith, J. Tight regulation, modulation, and high-level expression by vectors containing the arabinose PBAD promoter. *J.Bacteriol.* **177**, 4121–4130 (1995).
4. Bordes, P. *et al.* SecB-like chaperone controls a toxin-antitoxin stress-responsive system in Mycobacterium tuberculosis. *Proceedings of the National Academy of Sciences of the United States of America* **108**, 8438–43 (2011).
5. Bordes, P. *et al.* Chaperone addiction of toxin-antitoxin systems. *Nat Commun* **7**, 13339 (2016).
6. Genevaux, P. *et al.* In vivo analysis of the overlapping functions of DnaK and trigger factor. *EMBO Rep.* **5**, 195–200 (2004).
7. van Kessel, J. C. & Hatfull, G. F. Recombineering in Mycobacterium tuberculosis. *Nat. Methods* **4**, 147–152 (2007).
8. Blumenthal, A., Trujillo, C., Ehrt, S. & Schnappinger, D. Simultaneous Analysis of Multiple Mycobacterium tuberculosis Knockdown Mutants In Vitro and In Vivo. *PLOS ONE* **5**, e15667 (2010).
9. Jumper, J. *et al.* Highly accurate protein structure prediction with AlphaFold. *Nature* **596**, 583–589 (2021).
10. Russell, R. B. & Barton, G. J. Multiple protein sequence alignment from tertiary structure comparison: assignment of global and residue confidence levels. *Proteins* **14**, 309–323 (1992).
11. Hebditch, M. & Warwicker, J. Web-based display of protein surface and pH-dependent properties for assessing the developability of biotherapeutics. *Sci Rep* **9**, 1969 (2019).

Supplementary uncropped scans:

Supplementary Fig.1c

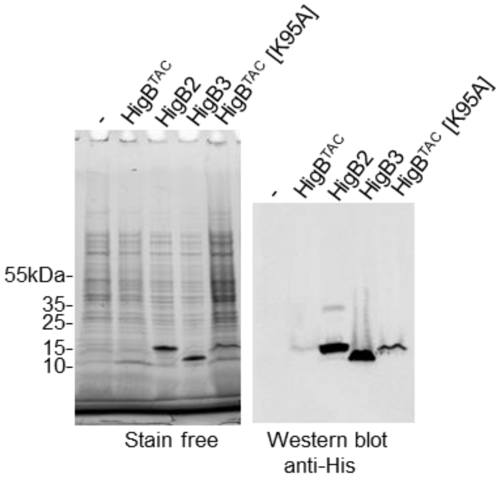

Supplementary Fig.1d

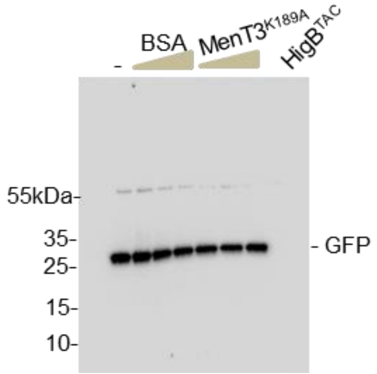

Supplementary Fig.3a

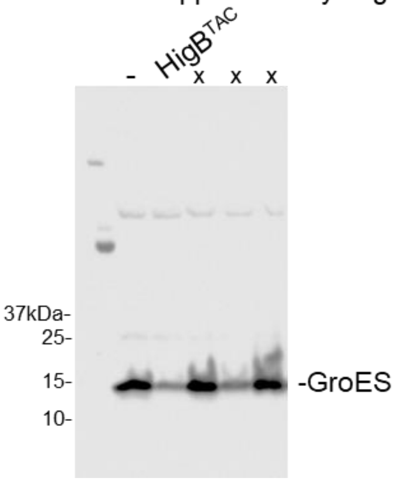

Supplementary Fig.3c

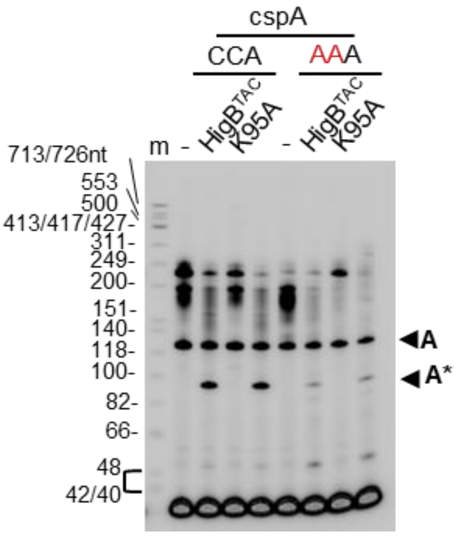

Supplementary Fig.3d

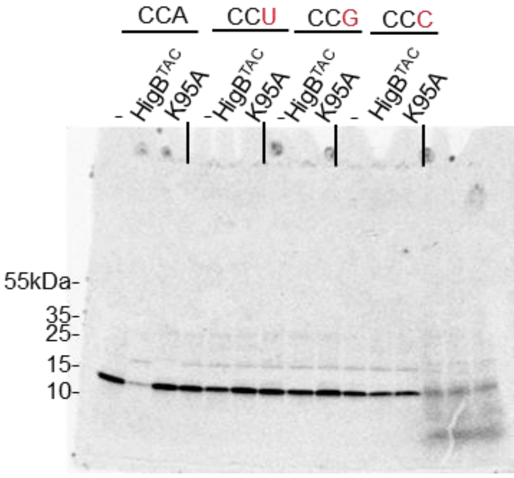

Supplementary Fig.3e

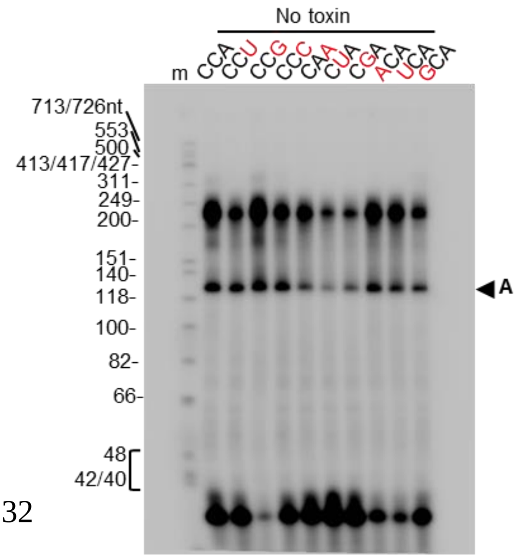

Supplementary Fig.4a

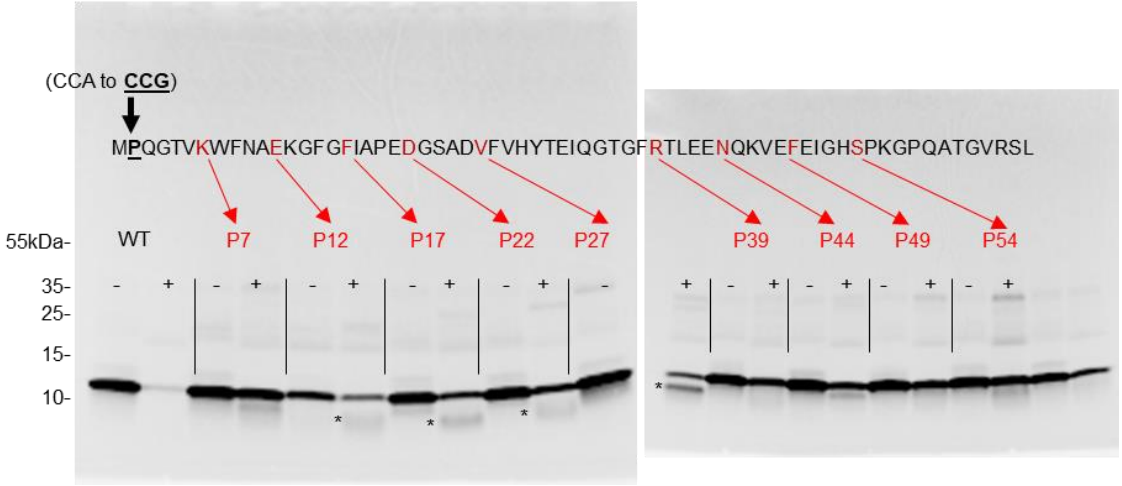

Supplementary Fig.4b

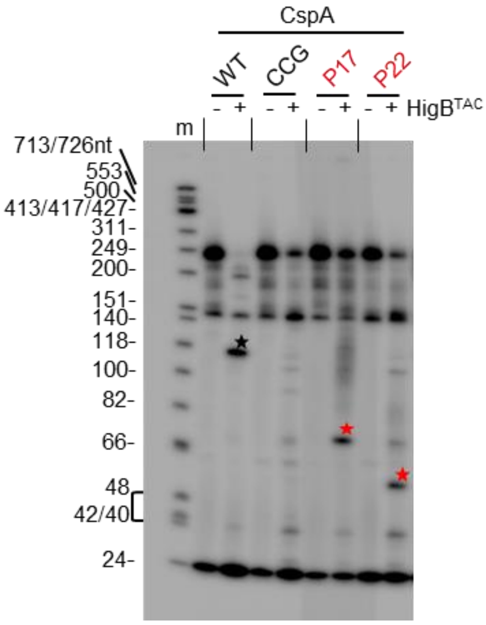

Supplementary Fig 8b

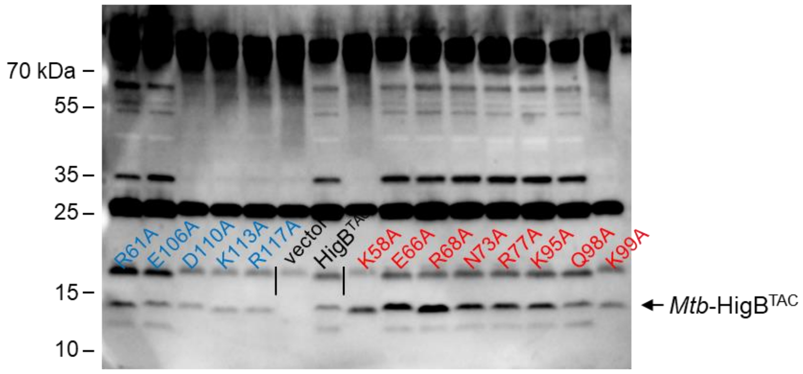

Supplement: Supplementary file 1 — Supplementary Information [file 41467_2022_30373_MOESM1_ESM.pdf]
