# Supplementary material for: Substrate recognition and cryo-EM structure of the ribosome-bound TAC toxin of Mycobacterium tuberculosis
Source: Nat Commun. 2022 May 12;13:2641. doi: 10.1038/s41467-022-30373-w (PMC9098466; doi:10.1038/s41467-022-30373-w)
Supplement: Supplementary file 3 — Description of Additional Supplementary Files [file 41467_2022_30373_MOESM3_ESM.pdf]

Description of Additional Supplementary Files

File name: Supplementary Data 1

Description: nEMOTE data
