# Supplementary material for: Substrate recognition and cryo-EM structure of the ribosome-bound TAC toxin of Mycobacterium tuberculosis
Source: Nat Commun. 2022 May 12;13:2641. doi: 10.1038/s41467-022-30373-w (PMC9098466; doi:10.1038/s41467-022-30373-w)
Supplement: Supplementary file 5 — Reporting Summary [file 41467_2022_30373_MOESM5_ESM.pdf]

nature portfolio

Corresponding author(s): Pierre Genevaux

Last updated by author(s): Mar 31, 2022

# Reporting Summary

Nature Portfolio wishes to improve the reproducibility of the work that we publish. This form provides structure for consistency and transparency in reporting. For further information on Nature Portfolio policies, see our Editorial Policies and the Editorial Policy Checklist.

## Statistics

For all statistical analyses, confirm that the following items are present in the figure legend, table legend, main text, or Methods section.

| n/a | Confirmed | |
|---|---|---|
| ☐ | ☒ | The exact sample size (*n*) for each experimental group/condition, given as a discrete number and unit of measurement |
| ☐ | ☒ | A statement on whether measurements were taken from distinct samples or whether the same sample was measured repeatedly |
| ☒ | ☐ | The statistical test(s) used AND whether they are one- or two-sided<br>*Only common tests should be described solely by name; describe more complex techniques in the Methods section.* |
| ☒ | ☐ | A description of all covariates tested |
| ☒ | ☐ | A description of any assumptions or corrections, such as tests of normality and adjustment for multiple comparisons |
| ☐ | ☒ | A full description of the statistical parameters including central tendency (e.g. means) or other basic estimates (e.g. regression coefficient) AND variation (e.g. standard deviation) or associated estimates of uncertainty (e.g. confidence intervals) |
| ☒ | ☐ | For null hypothesis testing, the test statistic (e.g. *F*, *t*, *r*) with confidence intervals, effect sizes, degrees of freedom and *P* value noted<br>*Give P values as exact values whenever suitable.* |
| ☒ | ☐ | For Bayesian analysis, information on the choice of priors and Markov chain Monte Carlo settings |
| ☒ | ☐ | For hierarchical and complex designs, identification of the appropriate level for tests and full reporting of outcomes |
| ☒ | ☐ | Estimates of effect sizes (e.g. Cohen's *d*, Pearson's *r*), indicating how they were calculated |

*Our web collection on statistics for biologists contains articles on many of the points above.*

## Software and code

Policy information about availability of computer code

| | |
|---|---|
| Data collection | Cryo-EM images were recorded at the Structural Biophysical Chemistry Platform of the IECB (Bordeaux, France) using SerialEM v3.6.14 and a Talos Arctica cryo-TEM (Thermo Fisher Scientific) operating at 200 kV and equipped with a field-emission gun. The diffraction datasets were collected on beamline MASSIF-3 (ID30-A3) at the European Synchrotron Radiation Facility (ESRF, Grenoble, France). |
| Data analysis | For cryo-EM data, movies were corrected for the effects of drift and beam-induced motion using MotionCor2 software (Zheng et al, 2017). Contrast transfer function (CTF) parameters were estimated using Gctf software (Zhang, 2016). Electron micrographs showing signs of drift or astigmatism were discarded, resulting in a dataset of 5,178 movies. Particles were semi-automatically selected in Cryosparc (Punjani et al, 2017). This resulted in the selection of 284,820 particles. All subsequent data processing was performed using RELION (Zivanov et al, 2018). SerialEM software was used to automatically record movies. The different molecules were manually adjusted in their respective multi-body maps using COOT (Casañal et al, 2020). The final atomic model was further improved by real-space refinement against the consensus maps using Phenix. The model quality was evaluated with MolProbity and the remaining analysis and the illustrations were done using UCSF-Chimera (Pettersen et al, 2004).<br>For the cristal structure, all data were indexed, integrated and scaled using XDS (Kabsch, 2010) and the CCP4 software suite was used for subsequent crystallographic calculations. The structures was solved by molecular replacement using PHASER (McCoy et al, 2007) and Iterative cycles of manual model building in COOT (Emsley et al, 2010) and refinement procedures using REFMAC (Murshudov et al, 2011) were applied until convergence.<br>For the Bioinformatic analysis, raw sequencing reads were first filtered and trimmed using the emoteStepI method from a Perl program called EMOTE-conv (Yasrebi & Redder, 2016). Plots are performed in R (version 3.4.4, running under Ubuntu 14.04.6) and putative cut-site motif was plotted with the R package ggseqlogo (version 0.1).<br>The software versions used for the Cryo-EM are :MotionCor2 v1.0.6; Gctf v1.18; Cryosparc v2.12; Relion v3.1.3; Resmap v1.1.4; molprobity v4.5.1; Coot v0.9.5 ; Phenix v1.18.2; UCSF-Chimera v1.13.1; serialEM v3.8.<br>The software used for X-ray: XDS Version Jan 31, 2020; CCP4 software suite Version 7.0.078; PHASER Version 2.8.3; COOT Version 0.9.6; REFMAC Version 5.8.0258 |

REFMAC Version 5.8.0258

For manuscripts utilizing custom algorithms or software that are central to the research but not yet described in published literature, software must be made available to editors and reviewers. We strongly encourage code deposition in a community repository (e.g. GitHub). See the Nature Portfolio guidelines for submitting code & software for further information.

## Data

Policy information about availability of data

All manuscripts must include a data availability statement. This statement should provide the following information, where applicable:

- Accession codes, unique identifiers, or web links for publicly available datasets
- A description of any restrictions on data availability
- For clinical datasets or third party data, please ensure that the statement adheres to our policy

All of the data supporting the findings of this study are available. The electron density maps and structure models are deposited in the EMDB and PDB under the following accession codes, respectively: 7AWK [http://doi.org/10.2210/pdb7awk/pdb] for the crystal structure of the M. Tuberculosis HigBTAC [K95A] toxin alone; and EMD-12261 [https://www.ebi.ac.uk/pdbe/entry/emdb/EMD-12261] and 7NBU [http://doi.org/10.2210/pdb7nbu/pdb] for the toxin and its target mRNA on the translating E. coli ribosome.

# Field-specific reporting

Please select the one below that is the best fit for your research. If you are not sure, read the appropriate sections before making your selection.

☒ Life sciences ☐ Behavioural & social sciences ☐ Ecological, evolutionary & environmental sciences

For a reference copy of the document with all sections, see nature.com/documents/nr-reporting-summary-flat.pdf

# Life sciences study design

All studies must disclose on these points even when the disclosure is negative.

| | |
|---|---|
| Sample size | No sample-size calculations were performed. For the cryo-EM, data were collected for at least one day, until the number of particles was sufficient for obtaining a high-resolution structure, as per standard single-particle analysis. Sample size was determined for obtaining enough particles to reconstruct high quality 3D map. |
| Data exclusions | Electron micrographs showing signs of drift or astigmatism were discarded. Particles in poorly resolved 2D/3D classes were excluded as per standard single-particle analysis procedures. No data were excluded from the other analyses. |
| Replication | All findings described here were confirmed by repeating the experiments (see figure legends) and, when possible/applicable, by performing distinct experiments to support the same experimental finding. For the stuctural data, multiple rounds of refinement were performed and they converged to the same structures. |
| Randomization | The single particle analysis was performed as per standard procedure, using Relion and the “gold standard” FSC procedure, meaning that during the map refinement the data set is divide into two random halves, and two set of model parameter are refined separately. Further than that the study does not involve to randomly allocate samples into experimental groups because it focus on a specific macromolecular complex. No randomization was necessary in our study, which concerns characterization of a molecular mechanism that relied on structural data. |
| Blinding | Blinding was not relevant for our study, which contains molecular biology experiments, biochemical reactions and structures, focusing on a specific macromolecular complex.. |

# Reporting for specific materials, systems and methods

We require information from authors about some types of materials, experimental systems and methods used in many studies. Here, indicate whether each material, system or method listed is relevant to your study. If you are not sure if a list item applies to your research, read the appropriate section before selecting a response.

## Materials & experimental systems

| n/a | Involved in the study |
|---|---|
| ☐ | ☒ Antibodies |
| ☒ | ☐ Eukaryotic cell lines |
| ☒ | ☐ Palaeontology and archaeology |
| ☒ | ☐ Animals and other organisms |
| ☒ | ☐ Human research participants |
| ☒ | ☐ Clinical data |
| ☒ | ☐ Dual use research of concern |

## Methods

| n/a | Involved in the study |
|---|---|
| ☒ | ☐ ChIP-seq |
| ☒ | ☐ Flow cytometry |
| ☒ | ☐ MRI-based neuroimaging |

# Antibodies

Antibodies used: Primary anti-HigBTAC, anti-GFP, StrepMAB-Classic HRP, anti-His6-HRP, and Horseradish peroxidase conjugated mouse IgG secondary

Validation: Polyclonal anti-HigBTAC antibody (dilution 1:1000) previously validated for western blot of whole cell extracts and published in Bordes et al, 2016, Nature Comm; monoclonal anti-StrepMAB-Classic HRP from Iba life sciences, (1:30000) were validated for western blot application (https://www.iba-lifesciences.com/strepmab-classic-conjugate/2-1509-001); Polyclonal anti-His6-HRP ref PA1-983B-HRP from Invitrogen is validated for western blots (https://www.thermofisher.com/antibody/product/6x-His-Tag-Antibody-Polyclonal/PA1-983B-HRP); Monoclonal anti-GFP (MA5-15256) was valivated for western blot (https://www.thermofisher.com/antibody/product/GFP-Antibody-clone-GF28R-Monoclonal/MA5-15256).
